# SREBP-2 promotes stem cell-like properties and metastasis by transcriptional activation of c-Myc in prostate cancer

**DOI:** 10.18632/oncotarget.7331

**Published:** 2016-02-11

**Authors:** Xiangyan Li, Jason Boyang Wu, Qinlong Li, Katsumi Shigemura, Leland W.K. Chung, Wen-Chin Huang

**Affiliations:** ^1^ Uro-Oncology Research Program, Department of Medicine, Samuel Oschin Comprehensive Cancer Institute, Cedars-Sinai Medical Center, Los Angeles, California, USA; ^2^ Department of Pathology, Xijing Hospital, Fourth Military Medical University, Xi'an, Shaanxi, China; ^3^ Department of Urology, Kobe University Graduate School of Medicine, Chuo-Ku, Kobe, Japan

**Keywords:** SREBP-2, stemness, metastasis, c-Myc, prostate cancer

## Abstract

Sterol regulatory element-binding protein-2 (SREBP-2) transcription factor mainly controls cholesterol biosynthesis and homeostasis in normal cells. The role of SREBP-2 in lethal prostate cancer (PCa) progression remains to be elucidated. Here, we showed that expression of SREBP-2 was elevated in advanced pathologic grade and metastatic PCa and significantly associated with poor clinical outcomes. Biofunctional analyses demonstrated that SREBP-2 induced PCa cell proliferation, invasion and migration. Furthermore, overexpression of SREBP-2 increased the PCa stem cell population, prostasphere-forming ability and tumor-initiating capability, whereas genetic silencing of SREBP-2 inhibited PCa cell growth, stemness, and xenograft tumor growth and metastasis. Clinical and mechanistic data showed that SREBP-2 was positively correlated with c-Myc and induced c-Myc activation by directly interacting with an SREBP-2-binding element in the 5′-flanking c-Myc promoter region to drive stemness and metastasis. Collectively, these clinical and experimental results reveal a novel role of SREBP-2 in the induction of a stem cell-like phenotype and PCa metastasis, which sheds light on translational potential by targeting SREBP-2 as a promising therapeutic approach in PCa.

## INTRODUCTION

Prostate cancer (PCa) is the most common cancer among men in the Western world [1]. The mechanisms underlying lethal PCa progression are far from being completely understood, and it is critically important to reveal the molecular basis of PCa metastatic progression to improve intervention strategies for treating this deadly disease.

PCa stem cells (PCSCs) are a unique cell population residing in tumors, which are responsible for tumor initiation, relapse, metastasis and resistance to therapy [2-4]. PCSCs exhibit self-renewal ability and can regenerate tumorigenic progeny by regulating a number of developmental signaling pathways including PTEN/PI3K/Akt [5], p53 [6] and NF-κB [7] pathways as well as by expressing stemness-related genes such as c-Myc [8], aldehyde dehydrogenase 1A1 (ALDH1A1) [9], CD44 [10], NANOG [11] and SOX-2 [12], which participate in maintaining the stem cell characteristics of cancer cells. These markers and regulators of stemness have been implicated in PCa metastatic progression and offer potential therapeutic targets for the treatment of PCa [13, 14].

Sterol regulatory element-binding proteins (SREBPs, including SREBP-1 and -2) are a family of basic helix-loop-helix leucine zipper transcription factors that regulate genes involved in fatty acid, lipid and cholesterol biosynthesis and homeostasis [15-17]. We and others previously showed that elevated SREBP-1 expression was observed in PCa with adverse pathologic and clinical features [18, 19], including a LNCaP xenograft mouse model that underwent castration, suggesting the functional role of SREBP-1 in castration-resistant PCa (CRPC) progression. In addition, SREBP-1 induced PCa growth and progression through concerted activation of the metabolic signaling networks involving androgen receptor (AR), lipogenesis and oxidative stress [19]. However, the role of SREBP-2 in PCa progression and metastasis still remains unclear.

In this study, we determined the role and molecular mechanism of SREBP-2 in promoting PCa growth and metastatic progression. Overexpression of SREBP-2 was associated with advanced pathological grades, metastatic potential and poor patient outcomes. Mechanistic studies demonstrated that SREBP-2 promoted PCa cell growth and the self-renewal capability of a stem-like cell population *in vitro*, and tumor initiation and metastasis *in vivo*. Knockdown of SREBP-2 inhibited PCa cell growth and reversed the induction of stem-like features and metastasis *in vivo*, further suggesting that SREBP-2 is required for maintenance of PCa stem cell-like properties and metastasis. Furthermore, SREBP-2 was positively correlated with c-Myc and induced c-Myc activation by directly interacting with an SREBP-2-binding element in the 5′-flanking c-Myc promoter region to drive PCa stemness and metastasis. Taken together, these findings establish the molecular basis of SREBP-2-dependent cell proliferation, stemness maintenance and PCa metastasis and provide a rationale for targeting SREBP-2 as a novel and promising therapeutic approach in PCa.

## RESULTS

### Overexpression of SREBP-2 is associated with human PCa progression and poor clinical outcomes

**Figure 1:**
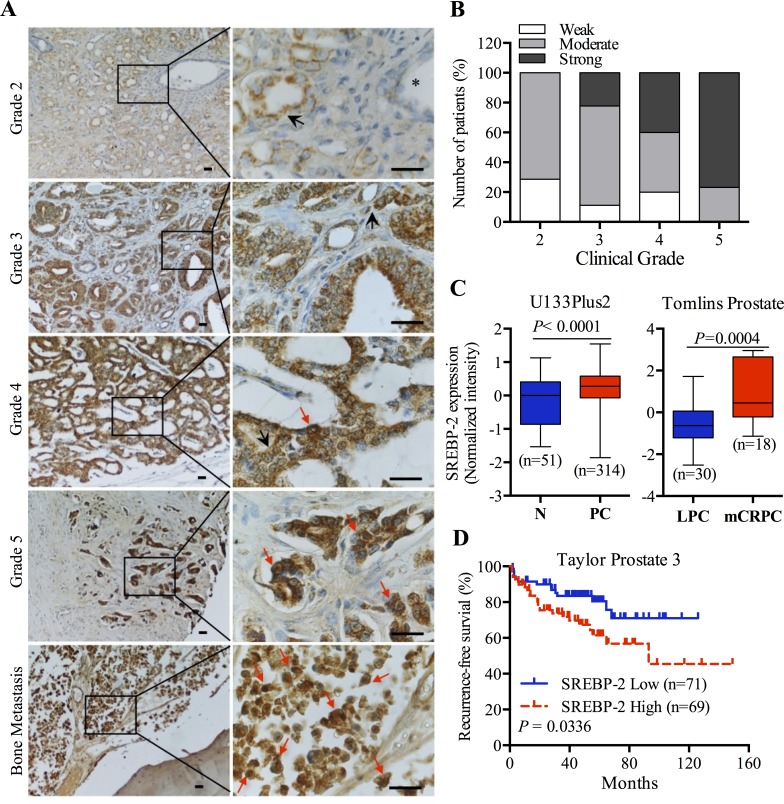
Overexpression of SREBP-2 is significantly associated with human PCa progression **A.** Representative images of SREBP-2 expression in a PCa tissue microarray (TMA) with different clinical grades and bone metastases. Absent or low expression of SREBP-2 was observed in normal prostate glands (black asterisk). The expression of SREBP-2 was increased in higher clinical grades of disease (cytoplasmic staining, black arrow; nuclear staining, red arrow). Scale bar = 20 μm. Detailed patient information is shown in Supplemental Table S1. **B.** Quantitative analysis of SREBP-2 staining showed a significant increase of protein level in higher clinical grades (+, weak; ++, moderate and +++, strong). **C.** Boxplot of SREBP-2 mRNA expression pattern in normal and PCa tissues from GENT (U133Plus 2) and Oncomine (Tomlins Prostate) databases. N, normal tissue; PC, prostate cancer tissue; LPC, local prostate cancer; mCRPC, metastatic castration-resistant prostate cancer. **D.** Correlation between high SREBP-2 expression and poor recurrence-free survival in PCa patients from Taylor Prostate 3 data set.

To define the clinical significance of SREBP-2 in human PCa, IHC staining was used to detect the expression of SREBP-2 in PCa patient specimens with different Gleason grades and bone metastasis. Compared to normal tissue (black asterisk), SREBP-2 protein was significantly upregulated in PCa and bone-metastatic tumor tissues (Figure 1A). Among all PCa patients, 87% (34 out of 39) of the cases showed elevated levels of SREBP-2, with only 5 cases weakly expressing SREBP-2 (Figure 1A; Supplementary Table S1). Moreover, SREBP-2 was highly expressed in both the cytoplasm (black arrow) and the nucleus (red arrow) in higher-grade and metastatic PCa specimens (Figure 1A). We also analyzed the correlation of SREBP-2 expression with clinical grades and Gleason scores within this cohort. A Chi-square test indicated that expression of SREBP-2 was significantly associated both with clinical grades (*P* = 0.0240) and Gleason scores (*P* = 0.0338) (Figure 1B; Table 1).

**Table 1:** Elevated expression of SREBP-2 is significantly associated with human PCa progression

Clinical and pathological features	Case	Weak (+, %)	Moderate (++, %)	Strong (+++, %)	*P* value*
Number of patients	39				
Age median (years)	66.0 (44-75)				
Clinical Grade					
2	7	2 (28.6)	5 (71.4)	0 (0.0)	
3	9	1 (11.1)	6 (66.7)	2 (22.2)	0.0240
4	10	2 (20.0)	4 (40.0)	4 (40.0)	
5	13	0 (0.0)	3 (23.1)	10 (76.9)	
Gleason Score					
6	7	2 (28.6)	5 (71.4)	0 (0.0)	
7	4	0 (0.0)	3 (75.0)	1 (25.0)	
8	12	1 (8.3)	7 (58.4)	4 (33.3)	0.0388
9	11	0 (0.0)	3 (27.3)	8 (72.7)	
10	5	0 (0.0)	2 (40.0)	3 (60.0)	
Pathological stage					
II	7	2 (28.6)	5 (71.4)	0 (0.0)	
III	20	3 (15.0)	7 (35.0)	10(50.0)	0.0915
IV	12	0 (0.0)	6 (50.0)	6 (50.0)	
Preoperative PSA (ng/ml)					
<10	8	0 (0.0)	2 (25.0)	6 (75.0)	
10-20	9	2 (22.2)	6 (66.7)	1 (11.1)	0.0973
>20	12	2 (16.7)	6 (50.0)	4 (33.3)	
Not available	10				

* Chi-square test.

Next, the DNA microarray data sets publically available at GENT and Oncomine were utilized to further confirm that the association of SREBP-2 expression with disease outcomes of PCa. As shown in Figure 1C (left panel), a survey of cancerous expression of SREBP-2 normalized with normal tissues, extracted from 6 independent data sets using the Affymetrix HG-U133 Plus 2 platform [20], reflected higher expression of SREBP-2 in PCa compared with normal tissues. Additionally, higher expression of SREBP-2 was detected in metastatic CRPC (mCRPC) samples compared with local PCa tissues in both Tomlins Prostate and Grasso Prostate data sets from the Oncomine database (Figure 1C, right panel; Supplementary Figure S1A). We subsequently investigated whether expression of SREBP-2 is correlated with the prognosis of PCa patients. Analysis of the two data sets, Taylor Prostate 3 and Grasso Prostate, revealed a trend towards poor prognoses, including reduced recurrence-free and overall survival time in PCa patients with high SREBP-2 expression compared to the low SREBP-2 expression group (Figure 1D; Supplementary Figure S1B). Taken together, these clinical data suggest that expression of SREBP-2 is positively associated with poor patient outcomes, further implying its critical role in PCa progression and metastasis.

### SREBP-2 promotes PCa cell proliferation, invasion and migration

**Figure 2:**
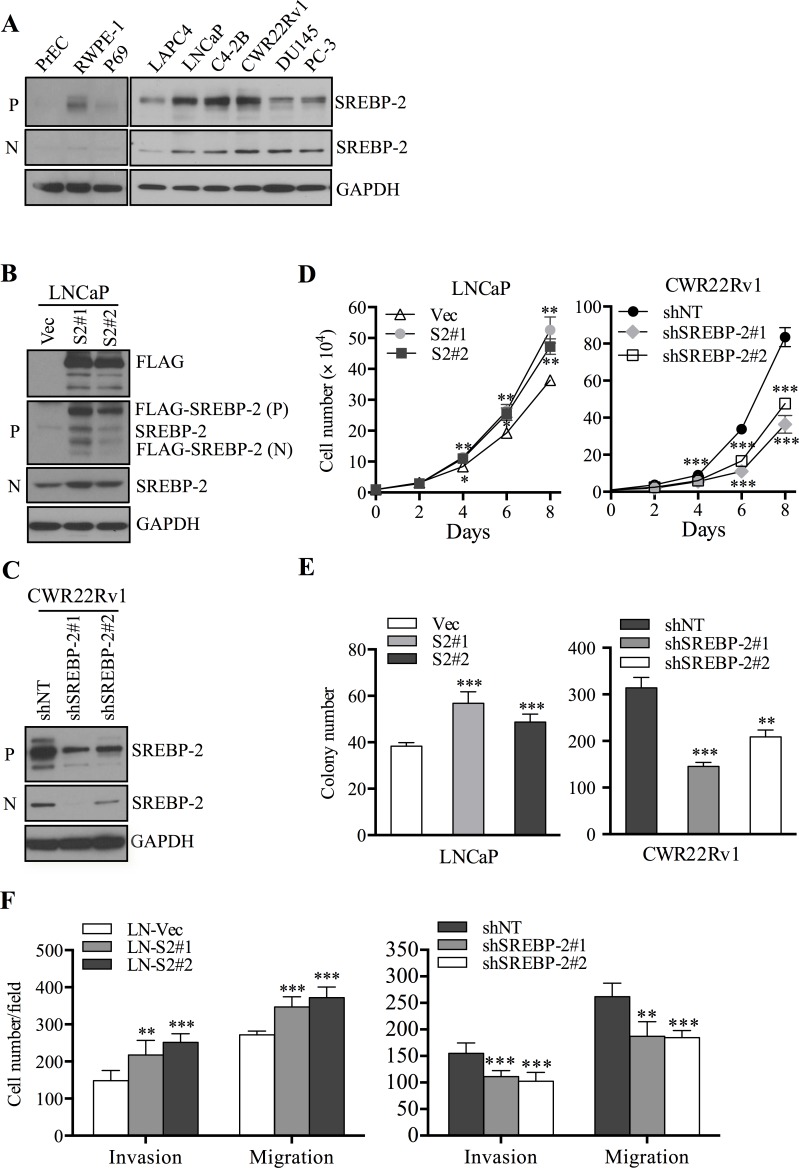
SREBP-2 promotes PCa cell proliferation, invasion and migration **A.** Western blot analysis of endogenous SREBP-2 expression in non-cancerous prostate cells (PrEC, RWPE-1 and P69) and PCa cells (LAPC4, LNCaP, C4-2B, CWR22Rv1, DU145 and PC-3). P, precursor form of SREBP-2; N, nuclear form of SREBP-2. **B.** Western blot analysis of SREBP-2 or FLAG expression level in LNCaP cells stably transfected with control (LN-Vec) or SREBP-2 vectors, LN-S2#1 (clone 1) and LN-S2#2 (clone 2). **C.** Western blot analysis of SREBP-2 expression in CWR22Rv1 cells stably transduced with non-target (shNT) or SREBP-2 shRNA lentiviral particles, shSREBP-2#1 (clone 1) and shSREBP-2#2 (clone 2). **D.** growth curves of LNCaP (control and SREBP-2-overexpression) or CWR22Rv1 (shNT and SREBP-2-knockdown) cells with genetically manipulated SREBP-2. Data were shown as the mean ± SD of three independent experiments. *P < 0.05, **P < 0.01, ***P < 0.001. **E.** colony formation of LNCaP or CWR22Rv1 cells with genetically manipulated SREBP-2. Data were shown as the mean ± SD of three independent experiments. **P < 0.01, ***P < 0.001. **F.** invasion and migration of SREBP-2-overexpressing LNCaP or SREBP-2-knockdown CWR22Rv1 and their respective control cells. Data represent the mean ± SD of three separate experiments. **P < 0.01, ***P < 0.001.

To investigate the role of SREBP-2 in human PCa cells, we first analyzed expression of endogenous SREBP-2 in a panel of normal prostatic and PCa cell lines. Consistent with the clinical results, expression of SREBP-2 protein in both precursor (125 kDa) and nuclear forms (68 kDa) was higher in PCa cell lines than that in normal prostatic cells (Figure 2A). Additionally, we found highly aggressive PCa cell lines, CWR22Rv1 and C4-2B cells with high level of SREBP-2 expression compared to that in low aggressive PCa cell lines, such as LAPC4 and LNCaP cells (Figure 2A). This suggests a potential role of SREBP-2 in mediating PCa cell growth and progression. On the basis of these findings, we used cell lines expressing either low endogenous (LAPC4 and LNCaP) or high basal (CWR22Rv1 and C4-2B) levels of SREBP-2 as models to address the hypothesis that SREBP-2 may be an important factor in promoting PCa growth and progression. Several cell clones with genetically manipulated SREBP-2 were obtained as follows: 1) two stable SREBP-2-overexpressing LNCaP clones (LN-S2#1 and LN-S2#2), and a control vector LNCaP cells (LN-Vec) (Figure 2B; Supplementary Figure S2A); 2) a LAPC4 clone transiently overexpressing SREBP-2 (LA-S2), and control empty vector LAPC4 cells (LA-EV) (Supplementary Figure S2B); 3) two stable clones of CWR22Rv1 cells with shRNA-mediated knockdown of SREBP-2 (shSREBP-2#1 and shSREBP-2#2), and a stable clone of control expressing non-targeting shRNA (shNT) (Figure 2C; Supplementary Figure S2C); and 4) stable clones of C4-2B cells subjected to SREBP-2 knockdown shRNA (shSREBP-2#1) and control shRNA (shNT) (Supplementary Figure S2D).

As expected, overexpression of SREBP-2 led to a significant increase of cell proliferation in LNCaP (LN-S2#1 and LN-S2#2) and LAPC4 (LA-S2) cells compared with their respective control cells (LN-Vec and LA-EV) (Figure 2D, left panel; Supplementary Figure S2E). Conversely, knockdown of SREBP-2 in CWR22Rv1 (shSREBP-2#1 and shSREBP-2#2) and C4-2B (shSREBP-2#1) cells reduced cell proliferation in comparison with their respective control cells (CWR22Rv1 shNT and C4-2B shNT) (Figure 2D, right panel; Supplementary Figure S2F). Furthermore, overexpression of SREBP-2 significantly increased the ability of LNCaP cells to develop anchorage-independent colonies (Figure 2E, left panel; Supplementary Figure S3A, top panel), while knockdown of SREBP-2 decreased the number of developed colonies in CWR22Rv1 and C4-2B cells (Figure 2E, right panel; Supplementary Figures S3A, bottom panel; and S3B). Additionally, the effects of SREBP-2 on cell invasion and migration were examined in these cells. Stably enforced expression of SREBP-2 led to significant increases LNCaP cell invasion and migration (Figure 2F, left panel; Supplementary Figure S3C, left panel). In contrast, the invasive and migratory capabilities of CWR22Rv1 and C4-2B cells were both obviously reduced after SREBP-2 knockdown (Figure 2F, right panel; Supplementary Figures S3C, right panel; and S3D). Taken together, these results suggest that SREBP-2 significantly enhances the growth and aggressive behaviors of PCa cells.

### SREBP-2 increases PCa stem cell population and prostasphere formation

**Figure 3:**
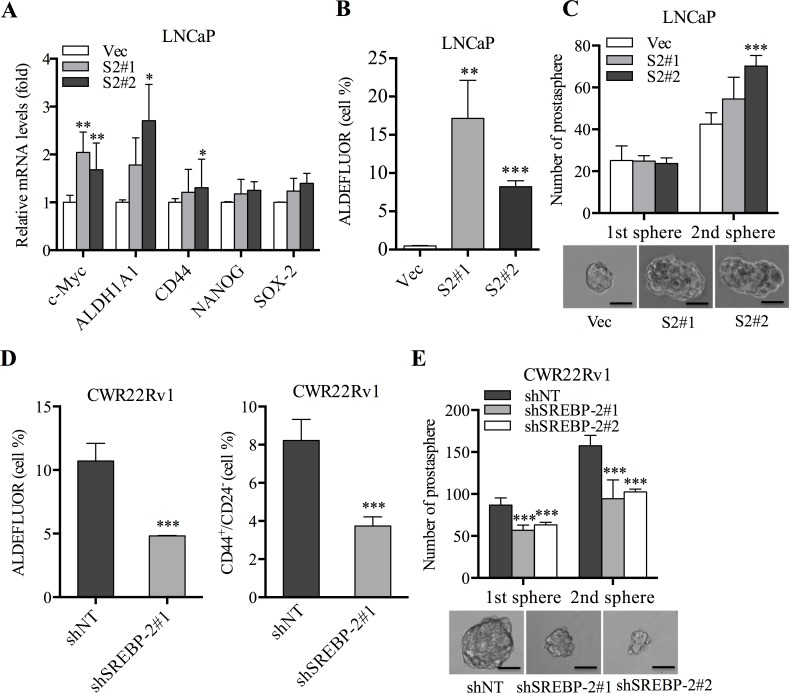
SREBP-2 induces PCa stem cell population increase and prostasphere formation **A.** Expression of c-Myc, ALDH1A1, CD44, NANOG and SOX-2 in LN-Vec, LN-S2#1 and LN-S2#2 cells was determined by qPCR. Data were normalized by β-actin and represent the mean ± SD of three independent experiments. *P < 0.05, **P < 0.01. **B.** Percentage of ALDH^high^ cells was assessed in LN-Vec, LN-S2#1 and LN-S2#2 cells by flow cytometry following the instructions of the ALDEFLUOR kit. Overexpression of SREBP-2 resulted in a significant elevation of the ALDH^high^ cell population compared with control cells. Data were shown as the mean ± SD of three independent experiments. **P < 0.01, ***P < 0.001. **C.** Primary and secondary prostasphere formation of LN-Vec, LN-S2#1 and LN-S2#2 cells. ***P < 0.001. Representative images of prostaspheres were shown on the bottom. Scale bar = 20 μm. **D.** Percentages of ALDH^high^ and CD44^+^/CD24^−^ cells in CWR22Rv1 shNT and shSREBP-2#1 cells were assessed by flow cytometry using the ALDEFLUOR kit, and anti-CD44 and anti-CD24 antibodies, respectively. Data were shown as the mean ± SD of from three independent experiments, ***P < 0.001. **E.** the numbers of primary and secondary prostaspheres of CWR22Rv1 shNT, shSREBP-2#1 and shSREBP-2#2 cells. ***P < 0.001.

The enrichment of PCSCs associated with aggressive progression, metastatic potentials and treatment resistance has been well defined [21, 22]. Here, we performed a series of experiments to explore the effect of SREBP-2 on stem cell population and prostasphere-forming ability in the established PCa cell clones. First, a group of stemness-related markers and regulators, including c-Myc, ALDH1A1, CD44, NANOG, and SOX-2, were determined in control and SREBP-2-overexpressing LNCaP cells by qPCR. Overexpression of SREBP-2 significantly increased expression of c-Myc, ALDH1A1 and CD44 expression, with slight increases of NANOG and SOX-2 expression in LNCaP cells (Figure 3A). We also confirmed these results by Western blot analysis where c-Myc and ALDH1A1 protein expressions were increased in SREBP-2-overexpressing LNCaP cells compared with control cells (Supplementary Figure S4A). Flow cytometric analysis further showed that overexpression of SREBP-2 resulted in a significant elevation of the ALDH^high^ stem cell subpopulation in LNCaP cells as compared to that in control cells (Figure 3B; Supplementary Figure S4B). Moreover, the prostasphere assay showed that overexpression of SREBP-2 in LNCaP cells developed larger prostaspheres than control (Figure 3C, bottom panel). Re-culturing prostaspheres harvested from the primary prostaspheres revealed that overexpression of SREBP-2 led to a significant increase in both the size and number of secondary prostaspheres compared with control prostaspheres (Figure 3C). In addition, overexpression of SREBP-2 in LAPC4 cells also increased the formation of primary and secondary prostaspheres as well as expression of c-Myc, ALDH1A1 and CD44 in comparison with control cells (Supplementary Figure S4C).

Conversely, decreases of c-Myc, ALDH1A1 and CD44 expression were observed after knockdown of SREBP-2 compared with that in control cells as determined by both qPCR and Western blot analyses (Supplementary Figure S5A). Furthermore, silencing of SREBP-2 expression led to reduction of the ALDH^high^ (Figure 3D, left panel; Supplementary Figure S5B) and CD44^+^/CD24^−^ subpopulations (Figure 3D, right panel; Supplementary Figure S5C) and also resulted in inhibition of primary and secondary prostaspheres, with smaller numbers and sizes of CWR22Rv1 cells (Figure 3E). Additionally, knockdown of SREBP-2 in C4-2B cells also supported the concept that SREBP-2 mediates stem cell phenotypes (Supplementary Figure S5D). Collectively, these data suggest that SREBP-2 is required for maintenance of stem cell population and phenotype through regulation of stemness-related genes in PCa cells.

### SREBP-2 is positively correlated with c-Myc expression and directly interacts with an SREBP-2-binding element in the 5′-flanking c-Myc promoter region

**Figure 4:**
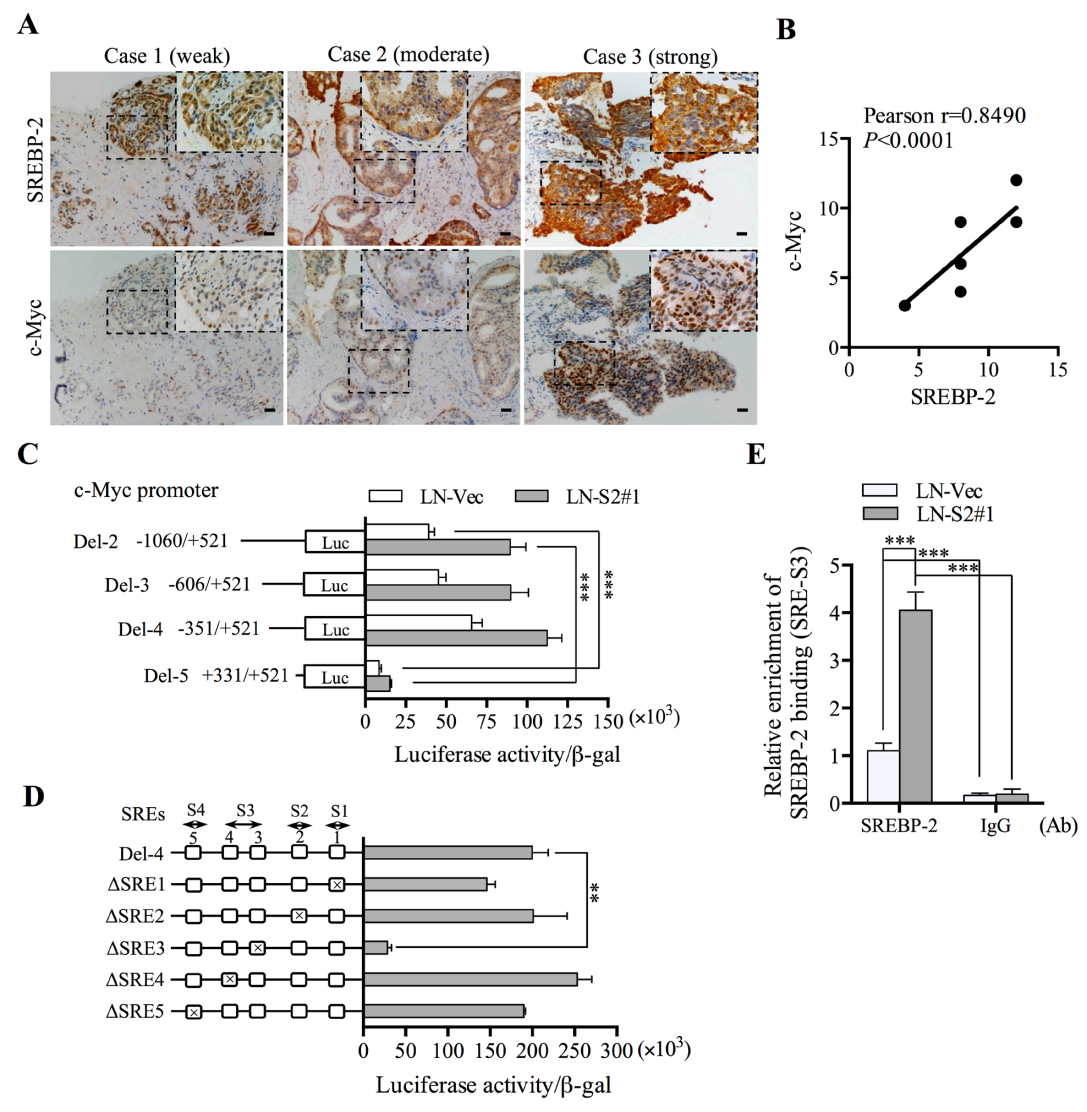
SREBP-2 is positively correlated with c-Myc expression in clinical PCa specimens and directly interacts with a SREBP-2-binding element in the 5′-flanking c-Myc promoter region **A.** IHC analysis was used for SREBP-2 and c-Myc expression in human PCa specimens. Representative images with weak, moderate or strong SREBP-2 and c-Myc expression were shown. The enlarged areas of metastatic tumor were indicated in the dashed rectangle. Scale bar = 20 μm. **B.** the correlation of SREBP-2 and c-Myc expression was analyzed using the Pearson's R correlation test. **C.** luciferase activity analysis of c-Myc promoter reporter constructs in LN-Vec and LN-S2#1 cells. ***P < 0.001. **D.** luciferase activity analysis of five putative SREBP binding site (SRE) deletion constructs and Del-4 c-Myc promoter construct in LN-S2#1 cells. Relative promoter activities were shown as the mean ± SD of three independent experiments. **P < 0.01. **E.** ChIP analysis of LN-Vec and LN-S2#1 cells immunoprecipitated by anti-SREBP-2 or IgG antibody (Ab) followed by qPCR analysis using a set of primers for SREBP-binding site, SRE-S3 in the c-Myc promoter. Data represent the mean ± SD of three independent experiments. ***P < 0.001.

Previous data showed that SREBP-2 affected stemness-associated gene expression, such as c-Myc. Interestingly, we found a similar expression pattern between SREBP-2 and c-Myc expression in PCa cell lines with different aggressive behaviors (Figure 2A; Supplementary Figure S6A). To further investigate the correlative expression of SREBP-2 and c-Myc in clinical PCa samples, we examined the co-expression pattern of SREBP-2 and c-Myc by IHC staining (Supplementary Table S2). Indeed, we observed a positive correlation between SREBP-2 and c-Myc expression (Figure 4A and 4B; Supplementary Table S3). Given the poor prognosis observed in patients with high level of SREBP-2 in Taylor Prostate 3 data set (Figure 1D), the correlation of SREBP-2 and c-Myc expression was further analyzed using the Pearson's R correlation test. A positive correlation of SREBP-2 and c-Myc expression was found, shown in Supplementary Figure S6B, which suggests the prognostic value of both SREBP-2 and c-Myc expression to predict disease progression in PCa patients.

Considering the innate feature of SREBP-2 as a transcription factor, we speculated that SREBP-2 might regulate c-Myc expression through a transcriptional regulatory mechanism. To test this possibility, we first used a series of c-Myc promoter luciferase reporter constructs with different promoter lengths (Del-2, −1060/+521; Del-3, −606/+521; Del-4, −351/+521; and Del-5, +331/+521) to examine c-Myc transcriptional activity in control and SREBP-2-overexpressing LNCaP cells. As shown in Figure 4C, higher c-Myc promoter activity was detected in SREBP-2-overexpressing (LN-S2#1) cells than that in control (LN-Vec) cells. Moreover, among all promoter constructs, a truncated Del-5 construct showed only background promoter activity compared to the others in both control and SREBP-2-overexpressing cells. These data suggest that the potential SREBP-2 *cis*-acting element(s) mediating c-Myc transcriptional activity may reside in the region between −351 and +331. To further identify the SREBP *cis*-acting element(s) in this region, we screened the sequence of this promoter fragment by an internet databank (TFSEARCH) and found 5 putative SREBP-binding sites as follows: SRE1 (+316/+326), SRE2 (+265/+275), SRE3 (−46/−36), SRE4 (−76/−67) and SRE5 (−151/−142) (Supplementary Figure S6C). Subsequently, we generated 5 deletion constructs, each harboring an individual deletion of a SRE (ΔSRE1, ΔSRE2, ΔSRE3, ΔSRE4 and ΔSRE5) and determined their luciferase activities in LN-S2#1 cells. Notably, the ΔSRE3 construct showed minimal promoter-luciferase activity compared to Del-4 and other SRE deletion constructs (Figure 4D). This result indicates that the SRE3 site (−46/−36) is likely to mediate c-Myc promoter activity regulated by SREBP-2 in PCa cells. In order to determine the *in vivo* association of SREBP-2 with these sites in the natural chromatin environment, we performed chromatin immunoprecipitation (ChIP) assays followed by qPCR analysis of all SREBP-2-binding sites in the c-Myc promoter in parental LNCaP cells. Only the enriched association of SREBP-2 protein with the SRE3 site was observed, but not other sites in the c-Myc promoter region (Supplementary Figure S6D). Additionally, a significant increase in binding affinity of SREBP-2 at the SRE3 site was further shown in SREBP-2-overexpressing LNCaP cells (LN-S2#1) compared with that in control cells (Figure 4E). Taken together, these clinical and mechanistic data suggest that SREBP-2 is positively correlated with c-Myc expression and mediates a functional SRE activation in the 5′-flanking region of c-Myc promoter in PCa cells.

### SREBP-2 induces stem cell-like properties through activation of c-Myc in PCa cells

**Figure 5:**
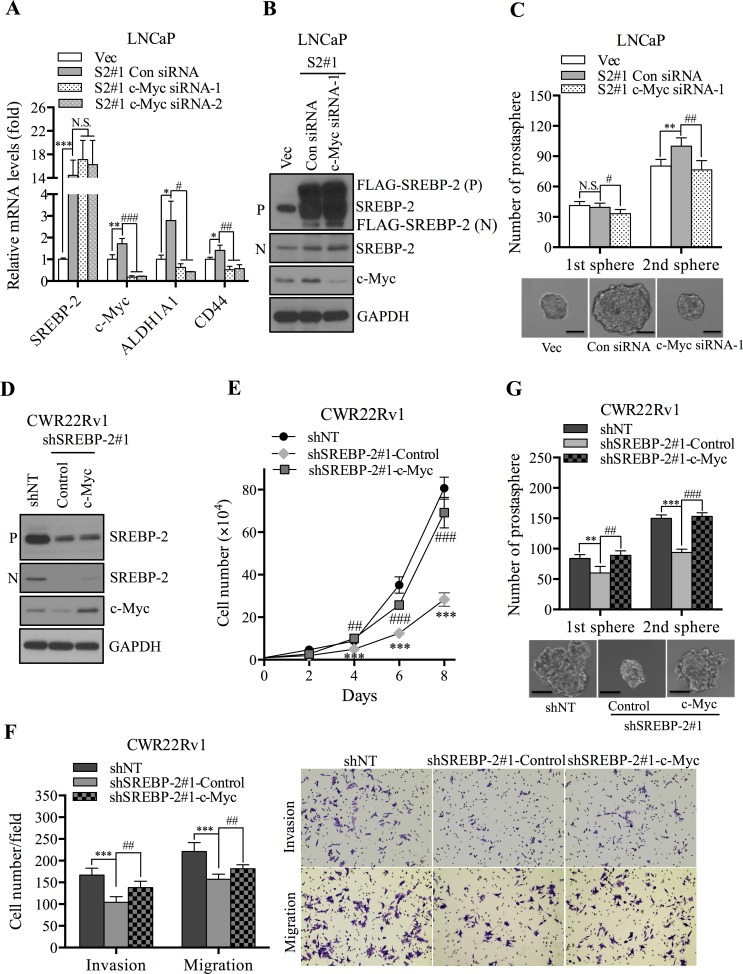
SREBP-2 induces stem-cell like properties through activation of c-Myc in PCa cells **A.** expression of SREBP-2, c-Myc, ALDH1A1 and CD44 mRNA in LN-Vec and LN-S2#1 cells transfected with control or c-Myc siRNAs (siRNA-1 and siRNA-2) was determined by qPCR. N.S., no significance; *P < 0.05, **P < 0.01, ***P < 0.001, compared with LN-Vec cells; #*P* < 0.05, ##*P* < 0.01, ###*P* < 0.001, compared with LN-S2#1 cells transfected with control siRNA. **B.** Western blot analysis of SREBP-2 and c-Myc in LN-Vec and LN-S2#1 cells transfected with control or c-Myc siRNA-1. **C.** numbers of primary and secondary prostasphere formation and representative images of LN-Vec and LN-S2#1 cells transfected with control or c-Myc siRNA-1. N.S., no significance; *P < 0.05, **P < 0.01, compared with LN-Vec cells; #*P* < 0.05, ##*P* < 0.01, compared with LN-S2#1 cells transfected with control siRNA. Scale bar = 20 μm. **D.** Western blot analysis of c-Myc and SREBP-2 in CWR22Rv1 shNT and shSREBP-2#1 cells transduced with control (shSREBP-2#1-Control) or c-Myc (shSREBP-2#1-c-Myc) expression vector retrovirus particles. **E.** growth curve of CWR22Rv1 shNT, shSREBP-2#1-Control and shSREBP-2#1-c-Myc cells. Data represent the mean ± SD from three independent experiments. ***P < 0.01, compared with shNT cells; ##*P* < 0.01, ###*P* < 0.001, compared with shSREBP-2#1-Control cells. **F.** Invasion and migration of CWR22Rv1 shNT, shSREBP-2#1-Control and shSREBP-2#1-c-Myc cells. ***P < 0.01, compared with shNT cells; ##*P* < 0.01, compared with shSREBP-2#1-Control cells. **G.** numbers of primary and secondary prostasphere formation and representative images of CWR22Rv1 shNT, shSREBP-2#1-Control and shSREBP-2#1-c-Myc cells. **P < 0.01, ***P < 0.01, compared with shNT cells. ##*P* < 0.01, ###*P* < 0.001, compared with shSREBP-2#1-Control cells. Scale bar = 20 μm.

As reported previously, c-Myc functions as a stemness regulator to maintain stem cell phenotypes. To identify the mechanism by which SREBP-2 regulates stem cell-like properties via c-Myc regulation, inhibition or restoration of c-Myc expression was conducted in SREBP-2-overexpressing or -knockdown PCa cells, respectively. We found that genetic silencing of c-Myc by specific siRNAs effectively repressed c-Myc expression in SREBP-2-overexpressing cells compared with that in control cells transfected with control siRNAs (Figure 5A and 5B). Moreover, the suppression of c-Myc decreased expression of ALDH1A1 and CD44 (Figure 5A) and impaired the ability of cells to form anchorage-independent colonies (Supplementary Figure S6E) compared with control cells. Furthermore, silencing of c-Myc significantly decreased both the number and size of primary and secondary prostaspheres compared with control cells (Figure 5C). If c-Myc were an important downstream mediator of SREBP-2, restoration of c-Myc expression would be expected to rescue the inhibition of cellular characteristics by SREBP-2 silencing. To test this hypothesis, we established control and c-Myc-overexpressing CWR22Rv1 cells with SREBP-2-knockdown background (shSREBP-2#1-Control and shSREBP-2#1-c-Myc, Figure 5D) and examined the effect of c-Myc reactivation on cell growth, aggressive behavior and prostasphere development in these cells. Overexpression of c-Myc induced cell growth (Figure 5E), and anchorage-independent colony formation (Supplementary Figure S6F) and also promoted the abilities of cell invasion and migration in SREBP-2-konckdown CWR22Rv1 cells (Figure 5F) compared with control cells. Additionally, restoration of c-Myc rescued the reduced prostasphere development by silenced SREBP-2 compared with that of control cells (Figure 5G). Collectively, these loss- and gain-of-function analyses suggest that c-Myc is a key downstream mediator of SREBP-2 in regulating stem cell-like properties in PCa cells.

### SREBP-2 promotes PCa tumorigenicity and metastasis *in vivo*

**Figure 6:**
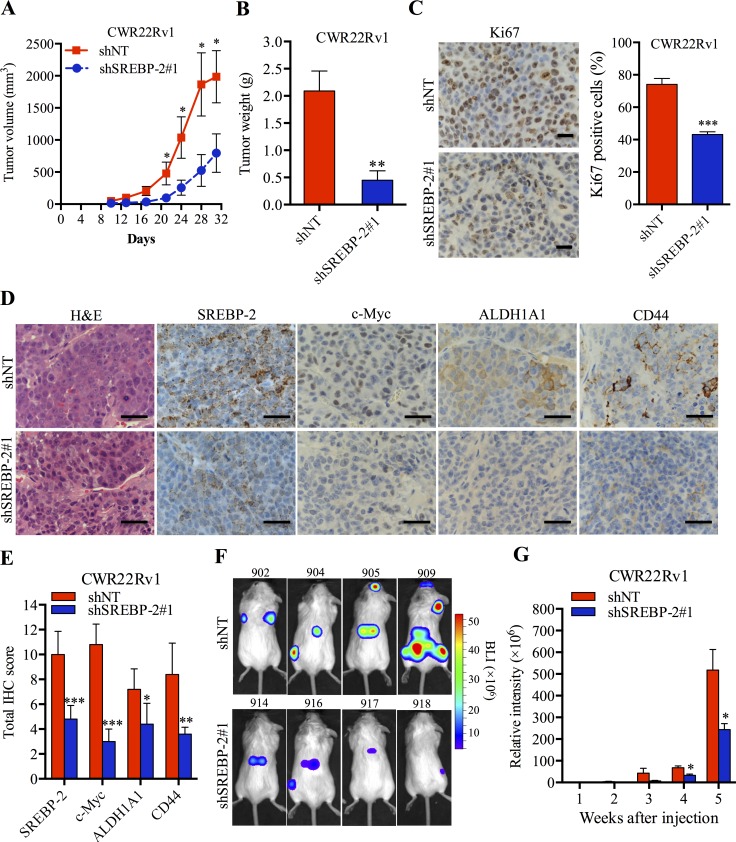
SREBP-2 promotes PCa tumorigenicity and metastasis *in vivo* **A.** CWR22Rv1 shNT and shSREBP-2#1 cells were subcutaneously injected into male nude mice (5 mice for each group). The tumor volumes of xenograft were measured and monitored at indicated time. *P < 0.05. **B.** The weights of subcutaneous CWR22Rv1 shNT and shSREBP-2#1 tumors 31 days after injection. **P < 0.01. Tumor images were shown in Supplementary Figure S7B. **C.** Representative images and the percentage of Ki67 positive cells in CWR22Rv1 shNT and shSREBP-2#1 tumor tissues. Scale bar = 20 μm, ***P < 0.001. **D.** H&E and IHC analysis of SREBP-2, c-Myc, ALDH1A1 and CD44 expression in CWR22Rv1 shNT and shSREBP-2#1 tumor tissues. Scale bar = 20 μm. **E.** Quantitative analysis of SREBP-2, c-Myc, ALDH1A1 and CD44 was performed and reported as total IHC score by assessing the intensity of each staining and the percentage of positive cells, shown as the mean ± SD from each group. *P < 0.05, **P < 0.01, ***P < 0.001. **F.** representative BLI images of mice bearing metastatic CWR22Rv1 shNT or shSREBP-2#1 tumors 5 weeks after intracardiac injection. **G.** normalized BLI signals of metastatic CWR22Rv1 shNT or shSREBP-2#1 tumor development over the course of 5 weeks. Data represent the mean ± SEM (*N* = 8). *P < 0.05.

Given that SREBP-2 was able to induce growth, invasion, migration and stemness of PCa cells as described above, we next examined whether these observations can be recapitulated *in vivo*. We established multiple PCa xenograft models in NOD.SCID, nude or SCID/Beige male mice. First, we established a subcutaneous transplantation model using a small number of control and SREBP-2-overexpressing LNCaP cells (2 or 20 × 10^3^ cells per site) in NOD.SCID mice. Enforced expression of SREBP-2 promoted LNCaP tumor engraftment *in vivo*, where successful engraftment was defined by higher tumor incidence initiated from fewer number of cells (e.g. 2 × 10^3^ cells; Supplementary Figure S7A). These results indicate that the stemness of LNCaP cells driven by SREBP-2 is acquired to initiate tumor development in mice. In line with the observation that inhibition of CWR22Rv1 cell growth *in vitro* by silencing of SREBP-2 (Figure 2D, right panel), a significantly reduced tumor growth rate and smaller tumors were observed in SREBP-2 knockdown CWR22Rv1 tumor xenografts compared with control tumors (Figure 6A, 6B and Supplementary Figure S7B). Ki67 staining of CWR22Rv1 xenograft tumor specimens further showed a 30% decrease of Ki67 positive cells in SREBP-2-knockdown tumors compared with the control group (Figure 6C). In addition, IHC and qPCR analyses revealed lower expression of SREBP-2, c-Myc, ALDH1A1 and CD44 in CWR22Rv1 tumors subjected to SREBP-2 silencing than that in control tumors (Figure 6D, 6E and Supplementary Figure S7C), consistent with the *in vitro* results (Supplementary Figure S5A). As c-Myc is a key downstream of SREBP-2, we also examined whether restoration of c-Myc expression could rescue the inhibition of tumor growth by silenced SREBP-2. We showed that overexpression of c-Myc was able to abolish the growth inhibition caused by silencing of SREBP-2 in a xenograft mouse model (Supplementary Figure S7D). Therefore, these studies suggest that SREBP-2 promotes PCa tumorigenicity, which is primarily mediated through direct targeting of c-Myc.

In light of the involvement of SREBP-2 in PCa tumor growth and development and its potential role in maintaining stemness and invasiveness of PCa cells as shown above, we further investigated whether SREBP-2 mediates PCa metastasis *in vivo*. To address this hypothesis, we performed intracardiac injections of luciferase-labeled control and SREBP-2-knockdown CWR22Rv1 cells in SCID/Beige mice to establish a rapid metastatic model and monitor tumor development of distant metastasis by weekly bioluminescence imaging. Compared to mice injected with control (shNT) cells which developed prevalent metastasis through the body, mice harboring SREBP-2-knockdown cells showed fewer gross metastatic sites as well as smaller metastatic tumors (Figure 6F and 6G; Supplementary Figures S8A and S8B). Tumor metastases in various organs, such as lung, adrenal gland and bone, were validated by routine necrotic procedures and histopathological analysis (Supplementary Figure S8B, bottom panel). We further analyzed the expression of SREBP-2 and stemness-related markers in metastatic tumor tissues, including adrenal gland and bone, from mice injected with control or SREBP-2-knockdown cells by IHC staining. Quantitative analysis revealed reduced expression of SREBP-2, c-Myc, ALDH1A1 and CD44 in both organs subjected to SREBP-2 silencing compared with control tumors (Supplementary Figure S8C), suggesting that the inhibition of metastasis by silencing SREBP-2 in PCa metastasis occurs through inhibiting c-Myc expression. Taken together, these animal studies provide significant evidence that SREBP-2 promotes PCa tumorigenicity and metastasis *in vivo* through direct activation of c-Myc.

## DISCUSSION

SREBPs have been implicated as important metabolic transcriptional factors in cancer. Studies have shown that the interconnections between cancer-associated PI3K/Akt/mTOR pathway and SREBP-mediated metabolic signaling network contribute to a number of critical cellular functions [23-25]. Mutant tumor suppressor p53 activates the mevalonate anabolic pathway and the YAP/TAZ pathway through transcriptional regulation of SREBP in breast cancer cells [26, 27]. Additionally, we reported that SREBP-1 induced PCa growth and progression via the coordinated activation of lipogenesis, reactive oxygen species (ROS)/oxidative stress and AR signaling [19]. However, the role of SREBP-2 in promoting lethal metastatic progression of PCa remains unclear. By analyzing the expression of SREBP-2 in the publicly available DNA microarray data sets and human PCa tissue microarrays, we observed significantly elevated SREBP-2 expression in PCa tissues with high Gleason scores and metastasis compared to low-score samples. Enforced expression of SREBP-2 enhanced growth, colony formation, migration and invasion in PCa cells and promoted tumorigenicity in a mouse model. Conversely, knockdown of SREBP-2 dramatically reduced PCa cell growth and aggressive behaviors *in vitro* and suppressed PCa metastasis to different distant organs, including lung, adrenal gland and bone *in vivo*. These findings reveal for the first time the critical role of SREBP-2 in promoting PCa growth and metastasis.

The existence of cancer stem cells may be involved in cancer initiation, progression and metastasis. In the present study, we proved the innovative concept that SREBP-2 induces stem cell genotypes and phenotypes in PCa cells. We employed different experimental approaches to characterize the effect of SREBP-2 on PCa cell stemness, including ALDEFLOUR assay, *in vitro* sphere-formation assay for the measurement of self-renewal capability and *in vivo* serial transplantation assay. SREBP-2 significantly increased ALDH^high^ cell populations displaying strongly elevated clonogenic, invasive and migratory behaviors [28]. We also found that SREBP-2 expression was positively correlated with ALDH1A1 level in human PCa tissues (Supplementary Figures S9A and S9B). Furthermore, SREBP-2 enhanced the percentage of CD44^+^/CD24^−^ subpopulation in PCa cells, which correlates with increased stem-like characteristics and predicts poor prognosis of PCa patients [2]. Consistently, SREBP-2 increased the development of prostaspheres and induced self-renewal capability in PCa cells. In animal studies, we demonstrated that SREBP-2-overexpressing LNCaP cells (only 2,000 cells) were able to develop subcutaneous xenograft tumors in mice while knockdown of SREBP-2 in CWR22Rv1 cells inhibited tumor growth and metastasis in the xenograft models along with decreased expression of stemness-related genes, including c-Myc, ALDH1A1 and CD44. These studies in aggregate provide significant evidence that SREBP-2 mediates the function and maintenance of stem cell genotypes and phenotypes in PCa cells, which may contribute to cancer aggressiveness and invasiveness.

c-Myc is an important transcription factor that has been extensively explored for its instrumental functions in regulation of growth of both normal and cancer cells [29], where it plays a key role in tumor initiation, progression and survival [30-32]. Silencing of c-Myc inhibited stem-like cell maintenance and tumorigenicity in PCa [33]. In this study, we showed a positive correlation between SREBP-2 and c-Myc expression in a panel of PCa cell lines and clinical specimens. Furthermore, we identified c-Myc as a novel target of SREBP-2. Specifically, we demonstrated that SREBP-2 directly interacted with c-Myc promoter through an SREBP-2 *cis*-acting element in the 5′-flanking region that controls c-Myc transcription. In order to prove that SREBP-2 induces stemness through c-Myc regulation, loss- and gain-of-function experiments were conducted in SREBP-2-manipulated PCa cells, which showed that RNAi-mediated c-Myc knockdown reverted stem cell-like properties induced by SREBP-2 and decreased expression of ALDH1A1 and CD44 in SREBP-2-overexpressing LNCaP cells. Conversely, the impaired self-renewal capability and reduced tumor growth in response to knockdown of SREBP-2 were relapsed by enforced expression of c-Myc in CWR22Rv1 cells. These results suggest that SREBP-2 controls c-Myc expression at the transcriptional level and this leads to regulation of stem cell features in PCa cells.

Stemness in cancer cells has been closely linked with epithelial-mesenchymal transition (EMT), which represents a series of phenotypic and behavioral changes in tumor cells, allowing their transition from indolent to virulent form with increased migratory, invasive and metastatic potential [34]. Through EMT, epithelial cancer cells lose cell polarity and cell-cell adhesion, and acquire mesenchymal characteristics. Interestingly, we noted a morphologic change of SREBP-2-overexpressing LNCaP cells compared to either control or parental cells, resulting in a mesenchymal-like, spindle-shaped form in culture (Supplementary Figure S10A). This SREBP-2-induced phenotypic switch of PCa cells is accompanied by markedly increased expression of mesenchymal-associated markers or regulators, including N-cadherin, Vimentin, Snail2 and ZEB1, and decreased production of an epithelial-specific marker, E-cadherin (Supplementary Figures S10B and S10C). Although the molecular basis of EMT has been well delineated in many types of cancer, including PCa, the molecular mechanisms by which SREBP-2 regulates EMT in PCa cells need to be further investigated.

Considering the key role of SREBP-2 in regulating cholesterol metabolism, we determined the expression of its canonical targets, including 3-methyl-glutaryl-CoA synthase 1 (HMGCS1), 3-hydroxy-3-methyl-glutaryl-CoA reductase (HMGCR), mevalonate kinase (MVK), mevalomate 5-pyrophosphate decarboxylase (MVD) and low-density lipoprotein receptor (LDLR) in PCa cells with genetically manipulated SREBP-2. Overexpression of SREBP-2 led to increases of HMGCS1, HMGCR, MVD and LDLR in LNCaP cells, while knockdown of SREBP-2 resulted in decreases of genes above in CWR22Rv1 cells (Supplementary Figures S11A and S11B). To further investigate whether stem cell-like properties induced by SREBP-2 in PCa cells could be contributed by cholesterol metabolism, simvastatin, an inhibitor of HMGCR blocking a primary and rate-limiting step for cholesterol biosynthesis, and exogenous cholesterol were used to treat PCa cells with genetically manipulated SREBP-2. The prostasphere forming ability and expression of stemness-related genes, including c-Myc, ALDH1A1 and CD44 were determined in these cell lines. Simvastatin treatment did not significantly affect prostasphere formation and expression of c-Myc, ALDH1A1 and CD44 in both control and SREBP-2 overexpressing LNCaP cells (Supplementary Figures S11C and S11D). Similarly, exogenous cholesterol did not induce stemness genotype and phenotype in both control and SREBP-2-knockdown CWR22Rv1 cells (Supplementary Figures S11E and S11F). These results provide evidence that SREBP-2-mediated stemness and c-Myc expression are not contributed by altered cholesterol metabolism in PCa cells, which is consistent with a previous study conducted in breast cancer cells [35].

In summary, our integrated studies in human clinical specimens, PCa cell lines and animal models uncovered a novel role of SREBP-2, a transcription factor conventionally considered as a regulator for cholesterol biosynthesis and homeostasis, in PCa lethal progression. Overexpression of SREBP-2 promoted PCa cell growth, self-renewal capability and metastasis *in vitro* and *in vivo,* which are reinforced by clinical observations that high SREBP-2 expression is significantly associated with metastasis and poor clinical outcomes of PCa patients. Through transcriptional regulation of c-Myc, SREBP-2 regulates PCa cell proliferation, aggressive behaviors and stemness. The SREBP-2/c-Myc axis is one of the critical driving forces promoting PCa growth, progression and metastasis. These findings provide insights into the potential for developing a novel therapy targeting the SREBP-2/c-Myc axis to treat metastatic PCa.

## MATERIALS AND METHODS

### Cell lines and culture conditions

Human prostate epithelial cells (PrEC) were purchased from Lonza Walkersville (Walkersville, MD) and cultured following the manufacturer's instructions. Normal/non-cancerous prostate cell lines (RWPE-1 and P69), PCa cell lines (LNCaP, CWR22Rv1, DU145 and PC-3) and HEK293T cells were originally purchased from American Type Culture Collection (ATCC, Manassas, VA). C4-2 and C4-2B cells were established by our laboratory [36, 37]. LAPC4 cells were acquired through a Material Transfer Agreement from University of California, Los Angeles (Los Angeles, CA) [38]. All cell lines were maintained in RPMI-1640 medium or T-medium (Life Technologies) supplemented with 10% FBS (Atlanta biological), 100 U/mL penicillin and 100 μg/mL streptomycin in a humidified 37°C incubator with 5% CO_2_.

### Plasmids and siRNA transfection and viral transduction

Human SREBP-2 cDNA was subcloned into p3XFLAG-myc-CMV-26 (Sigma-Aldrich) at NotI/XbaI restriction enzyme sites to generate p3XFLAG-SREBP-2 expression construct. LNCaP and LAPC4 cells were transfected with either p3XFLAG-SREBP-2 or empty vector as a control using Lipofectamine LTX Plus reagent (Life Technologies). For the SREBP-2 shRNA-mediated knockdown study, non-targeting control (pLKO.1, empty vector) or SREBP-2 shRNA lentiviral particles (Sigma-Aldrich) were used to infect CWR22Rv1 or C4-2B cells. In order to generate c-Myc-overexpressing PCa cell clones, retroviruses that carried either pWZL-Blast-Myc (Addgene) or control vector (pWZL-Blast) were used. For the silencing of c-Myc, cells were transfected with c-Myc siRNA-1, c-Myc siRNA-2 or control siRNA (Sigma-Aldrich) using DharmaFECT 2 transfection reagent (Fisher Scientific Dharmacon).

### Cell proliferation, colony formation, and progression assays

Cell proliferation, colony formation, cell invasion and migration assays were conducted as previously described [39, 40].

### Quantitative real-time PCR (qPCR) and Western blot analyses

qPCR and Western blot analyses were performed as previously described [39, 40]. The primer sequences are provided in the Supplementary Methods. Primary antibodies against SREBP-2 (ab72856, ab112046, ab30682; Abcam), FLAG (F3165; Sigma-Aldrich), ALDH1A1 (HPA002123; Sigma-Aldrich), c-Myc (5605; Cell Signaling Technology), CD44 (5640; Cell Signaling Technology), and GAPDH (5174; Cell Signaling Technology) were used.

### Prostasphere assay

Cells were plated in 24-well plates or 10-cm dishes with an ultra-low attachment surface (Corning) in serum-free medium supplemented with B-27 supplement (Life Technologies), EGF (20 ng/mL; Life Technologies), bFGF (10 ng/mL; Life Technologies), insulin (5 μg/mL; Sigma-Aldrich), and 0.4 % bovine serum albumin (Roche). Floating spheres were grown for 5 days, counted and dissociated with Accutase (Sigma-Aldrich) for secondary sphere expansion under the same conditions. Phase contrast images were obtained using the Nikon Eclipse Ti microscope (Melville).

### Flow cytometric analysis

ALDH enzymatic activity was determined by flow cytometry using the ALDEFLOUR^TM^ Kit (StemCell Technologies). Fluorochrome-conjugated monoclonal antibodies against human CD44 (103009; BioLegend) and CD24 (311105; BioLegend) were used for sorting the CD44^+^/CD24^−^ cell population by flow cytometry.

### Promoter deletion and luciferase reporter assay

Luciferase reporter plasmids containing different restriction fragments of human c-Myc promoter, including Del-2 (−1060/+521), Del-3 (−606/+521) and Del-4 (−351/+521) were obtained from Addgene. Putative SREBP-binding sites within the Del-4 promoter reporter construct were individually deleted by a QuikChange II Site-Directed Mutagenesis Kit (Agilent Technologies). DNA sequences of all plasmid constructs were verified by sequencing. The protocols for the transfection of promoter reporters and the measurement of luciferase activity were conducted as described previously [41].

### Chromatin immunoprecipitation (ChIP) analysis

ChIP analysis was performed as previously described [42] to determine the specific association of endogenous SREBP-2 protein with c-Myc promoter in parental, control vector and SREBP-2-overexpressing LNCaP cells by an EZ-ChIP^TM^-Chromatin Immunoprecipitation Kit (Millipore) following the manufacturer's instructions. Detailed methods and primer sequences are provided in the Supplementary Methods.

### Animal studies

All animal experiments were performed in accordance with the protocol approved by the Institution Animal Care and Use Committee at Cedars-Sinai Medical Center. Male athymic nude mice, NOD.SCID mice and SCID/Beige mice were purchased from Harlan Laboratories (Houston, TX) and maintained under specific pathogen-free conditions. PCa cells were resuspended in a total volume of 100 μL of PBS containing 50% Matrigel (BD Bioscience) and injected subcutaneously into the flanks of the mice. Tumor volume was measured and calculated using the formula V = 1/2 × length × width^2^. For the tumor metastatic model, 1 × 10^6^ luciferase-tagged CWR22Rv1 cells expressing either control or SREBP-2 shRNA were injected intracardially into 4-week-old SCID/Beige mice as described previously [43]. Bioluminescence imaging was performed weekly to monitor tumor metastasis using a Xenogen IVIS Spectrum Imaging System (PerkinElmer). At the end of the animal studies, tumor tissues and organs were harvested from euthanized mice and fixed with 4% formaldehyde for further histological analysis.

### Immunohistochemical (IHC) staining

PCa tissue microarrays (TMAs) were purchased from Imgenex (San Diego, CA). Histopathologic and clinical characteristics of all patients are shown in Supplementary Tables S1 and S2. IHC staining of human PCa specimens and tumor xenografts was conducted using primary antibodies against SREBP-2 (30682; Abcam), c-Myc (ab32072; Abcam), ALDH1A1 (HPA002123; Sigma-Aldrich), CD44 (5640; Cell Signaling Technology) or Ki67 (ab16667; Abcam) as previously described [40, 44]. Histopathologic characteristics and the quantitation of each staining sample were determined by pathologists at Cedars-Sinai Medical Center. Details are described in the Supplementary Methods.

### Statistical analysis

All quantitative results are expressed as the mean ± SD or mean ± SEM as indicated in figure legends. For Kaplan-Meier survival analysis, statistical significance was determined by the log-rank test. Comparisons were analyzed by unpaired Student's *t* test, Chi-square test and Pearson's R correlation test. *P* values of less than 0.05 were considered to be statistically significant.

## SUPPLEMENTARY MATERIAL FIGURES AND TABLES


