# Supplementary material for: SREBP-2 promotes stem cell-like properties and metastasis by transcriptional activation of c-Myc in prostate cancer
Source: Oncotarget. 2016 Feb 11;7(11):12869–84. doi: 10.18632/oncotarget.7331 (PMC4914327; doi:10.18632/oncotarget.7331)
Supplement: Supplementary file 1 [file oncotarget-07-12869-s001.pdf]

SREBP-2 promotes stem cell-like properties and metastasis by transcriptional activation of c-Myc in prostate cancer

Supplementary Material

Supplementary Methods

Supplementary References

Supplementary Figure Legends

Supplementary Figures S1-11

Supplementary Tables S1-4

Supplementary Methods

Cell proliferation, invasion and migration assays

For the growth curve assay, 1×10^4 cells were seeded in 24-well plates in triplicates. Cell numbers were counted over a period of 8 days. For the clonogenic assay, 500 or 1,000 cells were plated in 6-well plates. The developed colonies were stained with 1% crystal violet and counted after 14 or 21-day incubation. *In vitro* cell invasion or migration was determined by Boyden chambers (transwell inserts, 8 µm pore size) pre-coated with growth factor reduced Matrigel matrix (invasion assay, BD Biosciences) or collagen I (migration assay), respectively. 1.5×10^5 cells were seeded inside transwell inserts containing medium without serum for 48-hour incubation. The invading or migrating cells on the lower surface of the filters were fixed with 4% formaldehyde, stained using 1% crystal violet, and quantified by counting the number of stained cells in 5 independent fields.

Quantitative real-time PCR (qPCR)

The qPCR program was: a hot start at 95°C for 5 minutes followed by 40 cycles of denaturation at 95°C for 15 seconds, annealing at 60°C for 30 seconds and elongation at 72°C for 30 seconds. Data were analyzed by $2^{-\Delta\Delta CT}$ method (1), normalized to β-actin and represented as the average fold of three independent experiments. The primer sequences used for PCR analysis are listed below.

Gene	Forward (5’-3’)	Reverse (5’-3’)
SREBP-2	CCCCTGACTTCCCTGCTGCA	GCGCGAGTGTGGCCGGATC
c-Myc	TCAAGAGGCGAACACACAAC	GGCCTTTTCATTGTTTTCCA
ALDH1A1	AGTGCCCCTTTGGTGGATTC	AAGAGCTTCTCTCCACTCTTG
CD44	TCAGAGGAGTAGGAGAGAGGAAAC	GAAAAGTCAAAGTAACAATAACAGTGG
NANOG	ATGCCTCACACGGAGACTGT	AAGTGGGTTGTTTGCCTTTG
SOX-2	TTGCTGCCTCTTTAAGACTAGGA	CTGGGGCTCAAACTTCTCTC
HMGCS1	GAGGGCTTCGTGGGACACATA	GCCACTGGGATGGATCTTT
HMGCR	GTCATTCCAGCCAAGGTTGT	GGGACCACTTGCTTCCATTA
MVK	CCTTGTGGCTGGCGTCAGAAA	CGAGGGCATTCAGATGGTGCT
MVD	ACCACGGGGACACCACGGT	CCACACAGCAGCCACAAACTC
LDLR	CAACGGCTCAGACGAGCAAG	AGTCACAGACGAACTGCCGAGA
β-actin	CAAGGCCAACCGCGAGAAGATGAC	GCCAGAGGCGTACAGGGATAGCACA

Immunofluorescence staining

Cells were fixed with 10% formalin and permeabilized with 0.1% Triton X-100 in PBS. After washing with PBS, cells were blocked with 1% BSA for 1 hour. Anti-SREBP-2 (Abcam, ab30862) primary antibody was added and incubated at 4°C overnight. After washing twice in PBS, anti-rabbit IgG-FITC (sc-2090, Santa Cruz Technology) secondary antibody was added and incubated for 1 hour. For the examination of nuclear morphology, cells were stained with DAPI solution (1 μg/mL) for 10 minutes. Fluorescence images were visualized by fluorescence microscopy (Nikon Eclipe Ti) (2).

Flow cytometric analysis

ALDH activity was determined by flow cytometric analysis using the ALDEFLOUR^{TM} Kit (StemCell Technologies). Briefly, cells were harvested and incubated in ALDHEFLUOR assay buffer containing the substrate at 37°C for 30

minutes. Cells treated with diethylaminobenzaldehyde (DEAB, an ALDH inhibitor) were used to define sorting gates based on baseline fluorescence. For sorting a $CD44^+/CD24^-$ cell population, cells were dissociated with Accutase (Sigma-Aldrich) and washed twice in PBS with 0.5% FBS. Fluorochrome-conjugated monoclonal antibodies against CD44 (PE/Cy5, BioLegend) and CD24 (PE, BioLegend) were added to the cell suspension at appropriate concentrations recommended by the manufacturer and incubated at 4°C for 30 minutes in the dark. FACS was performed by a BD FACScan flow cytometer (BD Biosciences). Data were analyzed using FlowJo software (Tree Star).

Chromatin immunoprecipitation (ChIP) analysis

ChIP was conducted using an EZ-ChIP^{TM}-Chromatin Immunoprecipitation Kit (Millipore) following the manufacturer's instructions. Cells were fixed with 1% formaldehyde for 10 minutes and lysed in SDS lysis buffer supplemented with protease inhibitor cocktail. The chromatin was sheared by sonication to generate 200-1000 bp cross-linked DNA fragments. Immunoprecipitation was performed at 4°C overnight with anti-SREBP-2 antibody (Abcam) or IgG. After binding with protein A/G-Sepharose and washing with low- and high- salt wash buffer, the immunoprecipitated DNA was eluted and de-crosslinked by incubating at 65°C overnight. After treatment with RNase A and Proteinase K, the DNA was purified using spin columns and subsequently subjected to qPCR using different sets of primers specific to c-Myc promoter region encompassing different putative SREBP-2-binding sites, SRE-S1, SRE-S2, SRE-S3 and SRE-S4. One percent of chromatin prior to immunoprecipitation was saved as input and specific

enrichment of SREBP-2-bound DNA was calculated from three separate experiments. Primer sequences used for ChIP analysis are listed below.

Site	Forward (5'-3')	Reverse (5'-3')
SRE-S1	GAGCGAATAGGGGGCTTC	GCTCGGGTGTTGTAAGTTCC
SRE-S2	CCGGCTAGGGTGGAAGAG	GCTGCTATGGGCAAAGTTTC
SRE-S3	GGGTTCCCAAAGCAGAGG	GGTCCTCAGCCGTCCAGA
SRE-S4	CATGCGGCTCTCTTACTCTG	CCCTCTGCTTTGGGAACC

Histology and immunohistochemical (IHC) staining

For histological examination, tumors or organs harvested from euthanized mice were fixed with 4% formaldehyde solution overnight, embedded in paraffin and cut into 5-μm-thick sections. The deparaffinized sections were stained with hematoxylin and eosin (H&E). IHC staining was conducted using primary antibodies against SREBP-2 (ab30682, 1:100; Abcam), c-Myc (ab32072, 1:50; Abcam), ALDH1A1 (HPA002123, 1:100; Sigma-Aldrich), CD44 (5640, 1:100; Cell Signaling Technology) or Ki67 (ab16667, 1:100; Abcam) as described previously (2). The total IHC score (mixed cytoplasmic/nucleic staining) was calculated as the value of the percentage of positive staining × staining intensity, ranged from 0 to 12 as reported previously (3-5). The percentage of positive staining was scored as follows: negative as "0", less than 25% of tumor cells as "1", 25-50% of tumor cells as "2", 50-75% of tumor cells as "3", or more than 75% of tumor cells as "4". The quantitative expression levels of SREBP-2, c-Myc, ALDH1A1 and CD44 were defined as follows: "+" (weak, score 1-4), "++" (moderate, score 5-8), or "+++" (strong, score 9-12). All scoring was performed by pathologists at Cedars-Sinai Medical Center.

Supplemental References

1. Livak KJ, Schmittgen TD. Analysis of relative gene expression data using real-time quantitative PCR and the 2(-Delta Delta C(T)) Method. Methods 2001; 25: 402-8.
2. Li X, Chen YT, Hu P, Huang WC. Fatostatin Displays High Anti-Tumor Activity in Prostate Cancer by Blocking SREBP-Regulated Metabolic Pathways and Androgen Receptor Signaling. Mol Cancer Ther 2014.
3. De Marzo AM, Knudsen B, Chan-Tack K, Epstein JI. E-cadherin expression as a marker of tumor aggressiveness in routinely processed radical prostatectomy specimens. Urology 1999; 53: 707-13.
4. ten Berge RL, Snijdewint FG, von Mensdorff-Pouilly S, et al. MUC1 (EMA) is preferentially expressed by ALK positive anaplastic large cell lymphoma, in the normally glycosylated or only partly hypoglycosylated form. J Clin Pathol 2001; 54: 933-9.
5. Koo CL, Kok LF, Lee MY, et al. Scoring mechanisms of p16INK4a immunohistochemistry based on either independent nucleic stain or mixed cytoplasmic with nucleic expression can significantly signal to distinguish between endocervical and endometrial adenocarcinomas in a tissue microarray study. J Transl Med 2009; 7: 25.

Supplementary Figure Legends

Supplementary Figure S1. Analysis of SREBP-2 expression in human PCa tissues (Grasso Prostate, Oncomine database). **A**, boxplot displayed expression pattern of SREBP-2 in local prostate cancer (LPC, n=59) and metastatic castrate-resistant prostate cancer (mCRPC, n=35). **B**, correlation between SREBP-2 expression and overall survival in PCa patients from the data set in **A**.

Supplementary Figure S2. Establishment of SREBP-2-overexpressing and -knockdown PCa cells and the effect of SREBP-2 on cell proliferation. **A**, immunofluorescence staining and qPCR analysis of SREBP-2 expression in LN-Vec (vector control), LN-S2#1 (SREBP-2-overexpressing clone 1) and LN-S2#2 (SREBP-2-overexpressing clone 2) cells. ** $P < 0.01$, *** $P < 0.001$. **B**, qPCR and Western blot analysis of SREBP-2 expression in LA-Vec (vector control) and LA-S2 (SREBP-2-overexpressing) cells. *** $P < 0.001$. GAPDH was used as a loading control. P, precursor form of SREBP-2; N, nuclear form of SREBP-2. **C**, qPCR analysis of SREBP-2 expression in CWR22Rv1 shNT (non-targeting shRNA), shSREBP-2#1 and shSREBP-2#2 (SREBP-2 shRNA) cells. *** $P < 0.001$. **D**, qPCR and Western blot analysis of SREBP-2 expression in C4-2B-shNT (non-targeting shRNA) and C4-2B-shSREBP-2#1 (SREBP-2 shRNA). *** $P < 0.001$. **E**, growth curve of LA-Vec and LA-S2 cells. Data represent the mean ± SD of three separate experiments. * $P < 0.05$. **F**, growth curve of C4-2B shNT and C4-2B shSREBP-2#1 cells. Data represent the mean ± SD of three separate experiments. * $P < 0.05$, ** $P < 0.01$.

Supplementary Figure S3. SREBP-2 increases colony formation, invasion and migration in PCa cells. **A**, developed colonies of LN-Vec, LN-S2#1 and LN-S2#2 cells (upper panel) and CWR22Rv1 shNT, shSREBP-2#1 and shSREBP-2#2 cells (lower panel) stained by crystal violet were photographed. **B**, developed colonies of C4-2B shNT and C4-2B shSREBP-2#1 cells were stained by crystal violet, counted and shown as the mean ± SD from three separate experiments. ***$P < 0.001$. **C**, representative images of invaded or migrated SREBP-2-overexpressing LNCaP cells (left panel; Vec, S2#1 and S2#2) and SREBP-2-knockdown CWR22Rv1 cells (right panel; shNT, shSREBP-2#1 and shSREBP-2#2). **D**, invasion or migration assays of C4-2B shNT and shSREBP-2#1 cells. Data was shown as the mean ± SD from three separate experiments. **$P < 0.01$, ***$P < 0.001$.

Supplementary Figure S4. SREBP-2 induces stem cell population and prostasphere formation in PCa cells. **A**, Western blot analysis for c-Myc and ALDH1A1 in LN-Vec, LN-S2#1 and LN-S2#2 cells. GAPDH was used as a loading control. **B**, ALDH activity of LN-Vec, LN-S2#1 and LN-S2#2 cells was assessed by flow cytometry using an ALDEFLUOR kit. DEAB was used to establish the baseline fluorescence of these cells and define the ALDEFLUOR-positive region. **C**, primary and secondary prostasphere formation and expression of c-Myc, ALDH1A1 and CD44 mRNA levels in LA-Vec and LA-S2 cells. Representative images were shown at the bottom and the numbers of primary and secondary prostaspheres were counted and shown as the mean ± SD from four separate experiments. ***$P < 0.01$. Scale bar = 20 μm.

Supplementary Figure S5. SREBP-2 silencing reduces stem cell population and prostasphere formation in PCa cells. **A**, qPCR and Western blot analysis of c-Myc, ALDH1A1 and CD44 expression in CWR22Rv1 shNT, shSREBP-2#1 and shSREBP-2#2 cells. The bar graphs show the mean ± SD from three independent experiments. *P < 0.05, ***P < 0.001. **B**, ALDH activity of CWR22Rv1 shNT and shSREBP-2#1 cells was assessed by flow cytometry using an ALDEFLUOR kit. **C**, representative plots of flow-cytometric analyses for CD44-PE/Cy5/CD24-PE stained CWR22Rv1 shNT and shSREBP-2#1 cells. **D**, numbers and representative images of primary and secondary prostaspheres and qPCR analyses of c-Myc, ALDH1A1 and CD44 in C4-2B shNT and shSREBP-2#1 cells. The bar graphs show the mean ± SD from three independent experiments. *P < 0.05, **P < 0.01, ***P < 0.001. Scale bar = 20 μm.

Supplementary Figure S6. SREBP-2 promotes PCa stem cell-like properties through transcriptional activation of c-Myc. **A**, Western blot analysis of c-Myc in various normal/non-cancerous prostate epithelial and PCa cell lines. The correlation of SREBP-2 (nuclear form, N-SREBP-2) and c-Myc in these cell lines was calculated by Pearson's R correlation test (right panel). **B,** scatter plots showing the correlation between SREBP-2 and c-Myc in the patients from Taylor Prostate 3 databases. **C**, schematic representation of the nucleotide sequences for 5 putative SREBP *cis*-acting sites in the 5'-flanking c-Myc promoter region (Del-4). **D,** ChIP analysis of LNCaP cells immunoprecipitated by anti-SREBP-2 or IgG antibody followed by qPCR using 4 sets of primers for the SREBP-2-binding sites, SRE-S1, S2, S3 and S4 in the c-Myc promoter. Input: 2% of total lysate. **P < 0.01, N.S., no significance. **E**, numbers and representative images of the developed

colonies of LN-Vec and LN-S2#1 cells transfected with control or c-Myc siRNA-1. Data were shown as the mean ± SD from three independent experiments. *$P < 0.05$, compared with LN-Vec cells; ## $P < 0.01$, compared with LN-S2#1 control siRNA cells. **F**, numbers and representative images of the developed colonies of CWR22Rv1 shNT and shSREBP-2#1 cells transduced with control (shSREBP-2#1-Control) or c-Myc expression (shSREBP-2#1-c-Myc) vector. Data represented as the mean ± SD collected from three independent experiments, ***$P < 0.001$, compared with shNT cells. ###$P < 0.001$, compared with shSREBP-2#1-Control cells.

Supplementary Figure S7. SREBP-2 plays an important role in PCa tumorigenicity through activation of c-Myc *in vivo.* **A**, xenograft tumors and tumor incidence derived from LN-Vec and LN-S2#1 cells in NOD.SCID mice 120 days after subcutaneous inoculation. Cells (2K or 20K cells) were resuspended in a total volume of 100 μL PBS containing 50% Matrigel and subcutaneously injected into the flanks of the mice. **B**, representative images of subcutaneous CWR22Rv1 shNT and shSREBP-2#1 tumors (31 days after the injection). **C**, qPCR analysis of SREBP-2, c-Myc, ALDH1A1 and CD44 expression in subcutaneous CWR22Rv1 shNT and shSREBP-2#1 tumors. Data were shown as the mean ± SD from each group (N = 4). *$P < 0.05$, ***$P < 0.001$. **D,** tumor growth after injection of CWR22Rv1 shNT, shSREBP-2#1-Control and shSREBP-2#1-c-Myc cells (N = 5 mice for each group) in nude mice was determined by measuring tumor size (the mean ± SEM) at the indicated time. *$P < 0.05$, compared with shNT tumor; #$P < 0.05$, compared with shSREBP-2#1-Control tumor (left panel). Tumor weight and the image from xenograft mouse model injected with CWR22Rv1 shNT, shSREBP-2#1-

Control and shSREBP-2#1-c-Myc cells were shown (right panel). ***P < 0.001, compared with shNT tumor; ##P < 0.01, compared with shSREBP-2#1-Control tumor.

Supplementary Figure S8. SREBP-2 promotes PCa metastasis *in vivo.* **A,** bar graphs showing the number of distant metastatic sites observed by luciferase signal in mice 5 weeks after intracardiac injection of CWR22Rv1 shNT and shSREBP-2#1 cells. *P < 0.05. **B**, summary of CWR22Rv1 shNT and shSREBP-2#1 tumor metastatic sites 5 weeks after intracardiac injection. SREBP-2 knockdown reduced the frequency and burden of PCa metastatic progression. Representative H&E staining of lung, adrenal gland and tibia tissues harvested from mice bearing CWR22Rv1 shNT tumors. The enlarged areas of metastatic tumor are indicated in the dashed rectangle. Scale bar = 20 μm. **C**, IHC analysis of SREBP-2, c-Myc, ALDH1A1 and CD44 expressions in adrenal gland and bone tissues from mice bearing metastatic CWR22Rv1 shNT or shSREBP-2#1 tumors. Quantitative analysis was reported as total IHC score by assessing the intensity of each staining and the percentage of positive cells, and shown as the mean ± SD from each group (bottom panel). Scale bar = 20 μm. *P < 0.05, **P < 0.01, ***P < 0.001.

Supplementary Figure S9. SREBP-2 expression was associated with ALDH1A1 in clinical PCa specimens. **A**, representative images showing weak, moderate or strong ALDH1A1 expression relative to SREBP-2 level (Fig. 4A, top panel). Scale bar = 20 μm. **B**, scatter plots showing the positive correlation between SREBP-2 and ALDH1A1 in PCa patients.

Supplementary Figure S10. SREBP-2 induces epithelial to mesenchymal transition (EMT) in LNCaP cells. **A**, representative phase-contrast images of LN-Vec, LN-S2#1 and LN-S2#2 cells. Scale bar = 20 μm. **B**, overexpression of SREBP-2 results in gain of mesenchymal markers (N-Cad, Vimentin, Snail2 and ZEB1) and loss of epithelial markers (E-Cad) in LN-Vec, LN-S2#1 and LN-S2#2 cells determined by qPCR. $^{*}P < 0.05$, $^{**}P < 0.01$, $^{***}P < 0.001$. **C**, Western blot analysis revealed increases in N-Cad and Vimentin and decrease in E-Cad with SREBP-2. GAPDH was used to show equal loading. N-Cad, N-cadherin; E-Cad, E-cadherin.

Supplementary Figure S11. Stem cell-like properties mediated by SREBP-2 are not through cholesterol metabolism in PCa cells. **A**, qPCR analysis of HMGCS1, HMGCR, MVD, and LDLR mRNA levels (left panel) and Western blot analysis of HMGCR expression (right panel) in control (LN-Vec) and SREBP-2-overexpressing LNCaP cells (LN-S2#1 and LN-S2#2). **B**, qPCR analysis of HMGCS1, HMGCR, MVK, MVD, and LDLR mRNA levels (left panel) and Western blot analysis of HMGCR expression (right panel) in control (shNT) and SREBP-2-knockdown CWR22Rv1 cells (shSREBP-2#1 and shSREBP-2#2). qPCR data were normalized to β-actin and represent the mean ± SD of three independent experiments. $^{*}P < 0.05$, $^{**}P < 0.01$, $^{***}P < 0.001$. **C**, primary and secondary prostasphere formation of LN-Vec and LN-S2#1 cells treated with vehicle or Simvastatin (10 μM) for 24 hours (top panel). Representative images of prostaspheres from one experiment were shown at the bottom. Scale bar = 20 μm. N.S., no significance. **D**, qPCR and Western blot analyses of SREBP-2 and stemness related markers (c-Myc, ALDH1A1 and CD44) in LN-Vec and LN-S2#1 cells treated with vehicle or Simvastatin

after 24-hour treatment. GAPDH was used as a loading control. N.S., no significance. **E**, primary and secondary prostasphere formation of CWR22Rv1 shNT and shSREBP-2#1 cells treated with additional cholesterol (10 μM) for 48 hours (top panel). Representative images of prostaspheres from one experiment were shown at the bottom. Scale bar = 20 μm. N.S., no significance. **F**, qPCR and Western blot analyses of SREBP-2 and stemness related markers in CWR22Rv1 shSREBP-2#1 cells treated with cholesterol for 48 hours. N.S., no significance.

Supplementary Figure S1

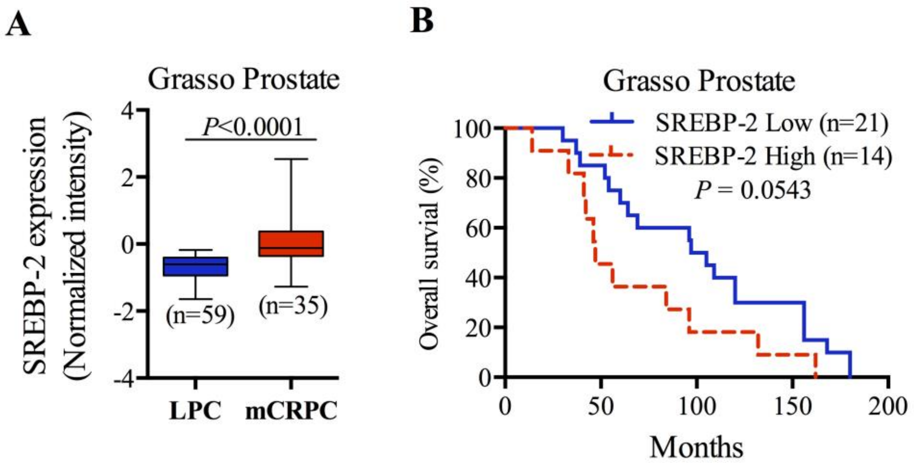

Supplementary Figure S2

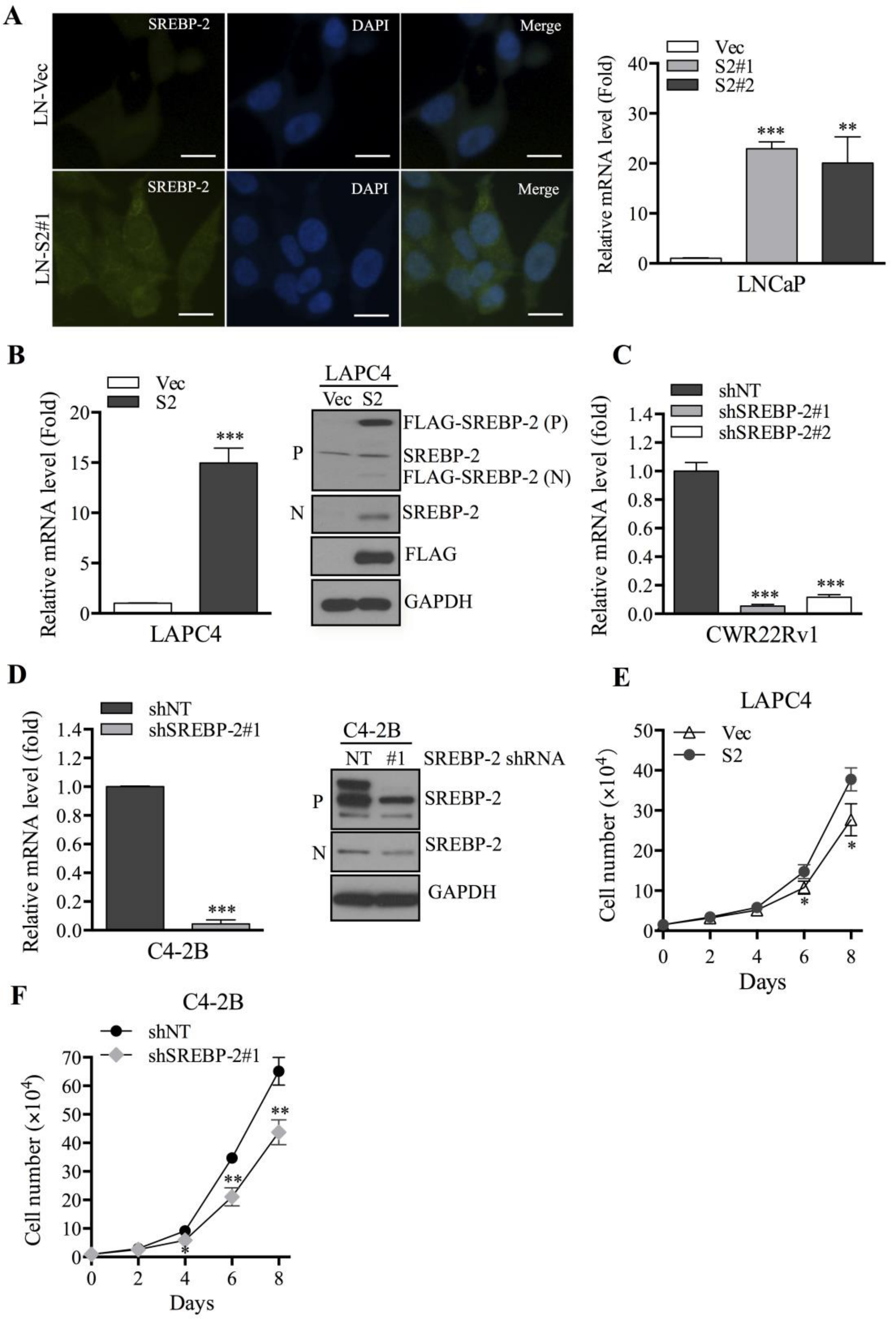

Supplementary Figure S3

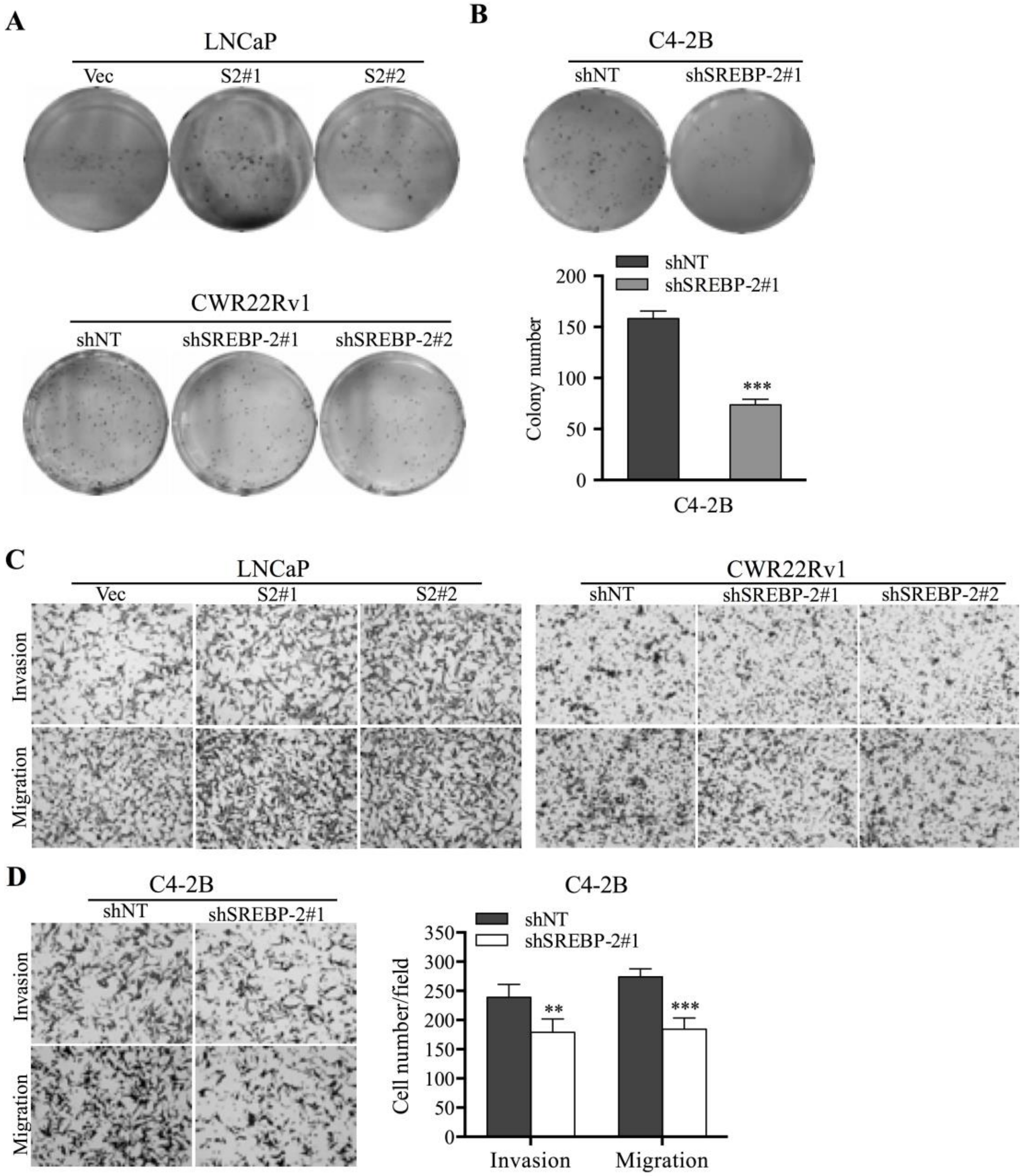

Supplementary Figure S4

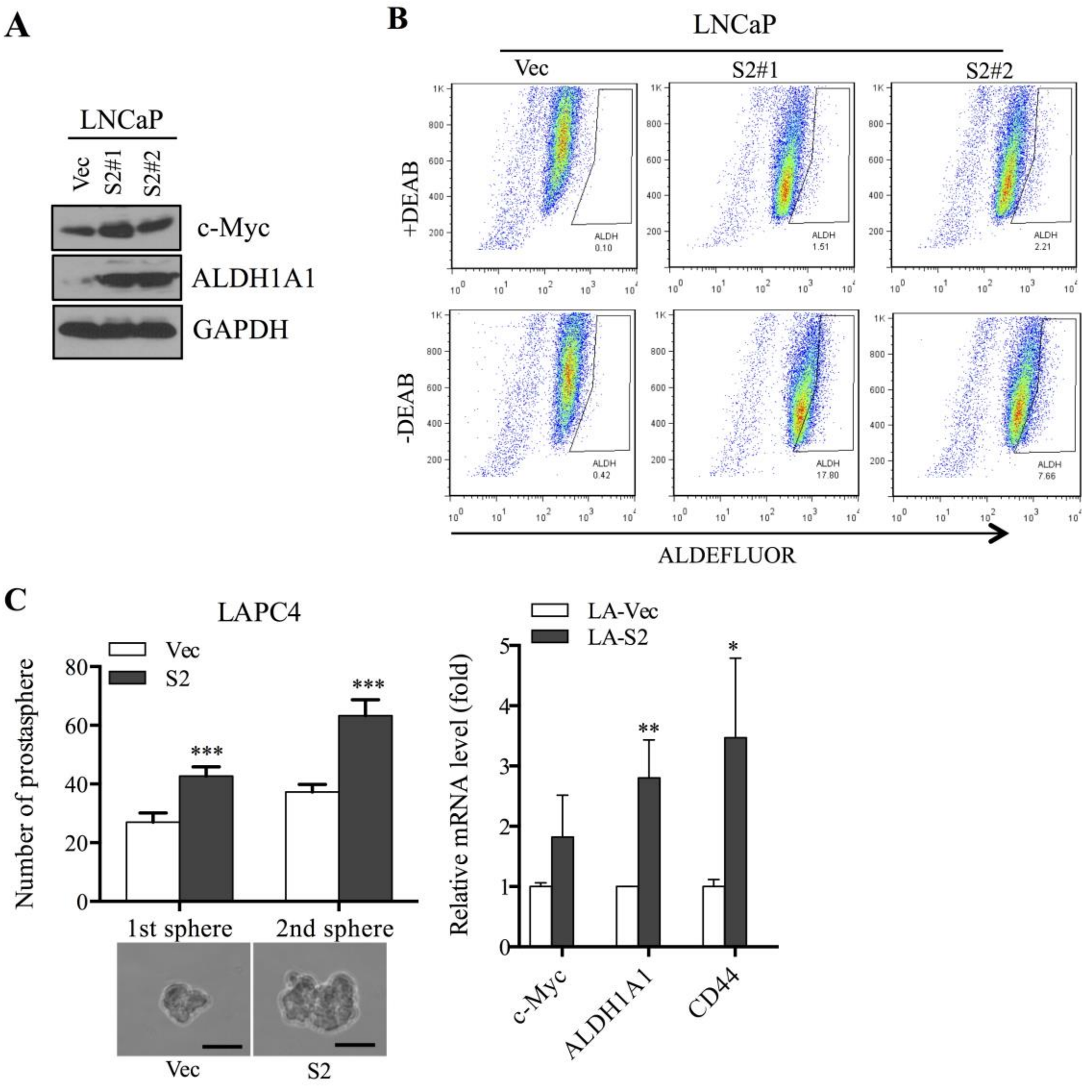

Supplementary Figure S5

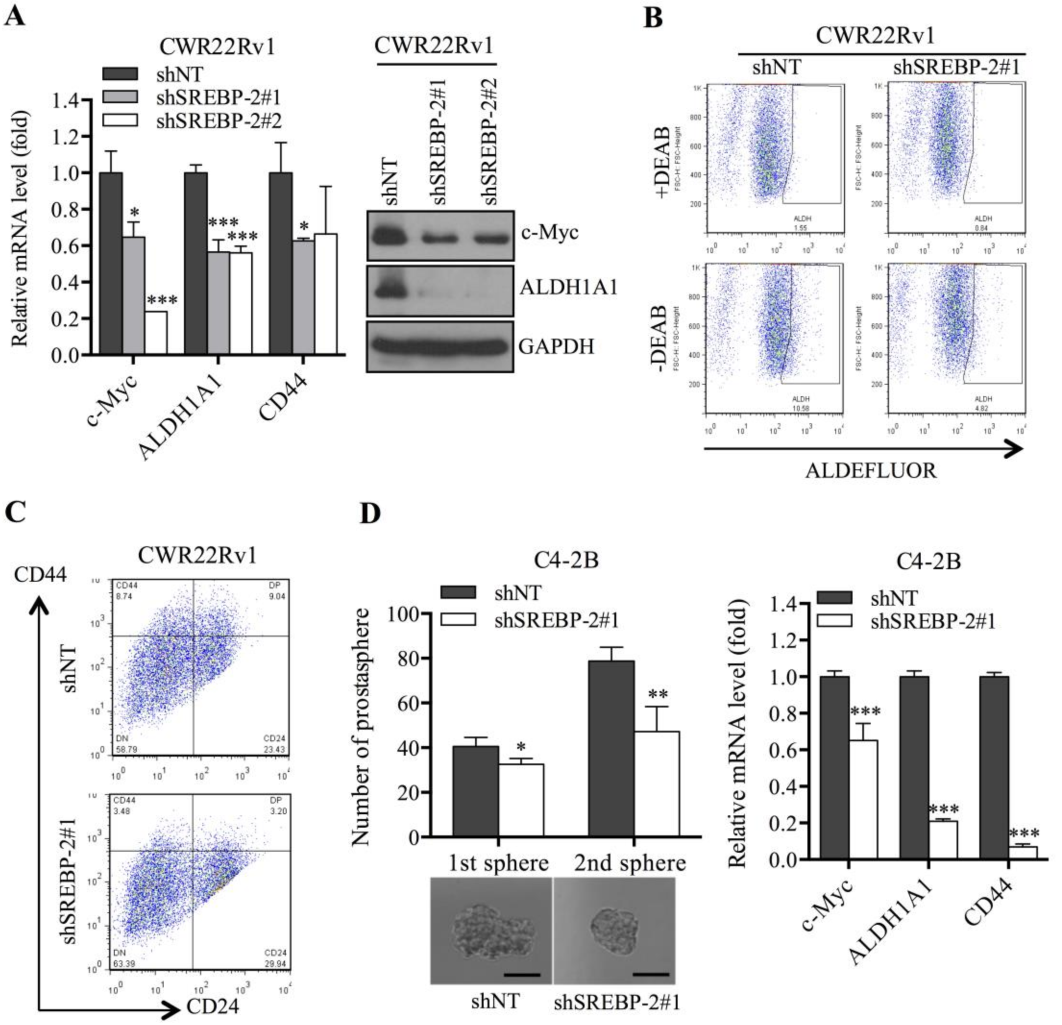

Supplementary Figure S6

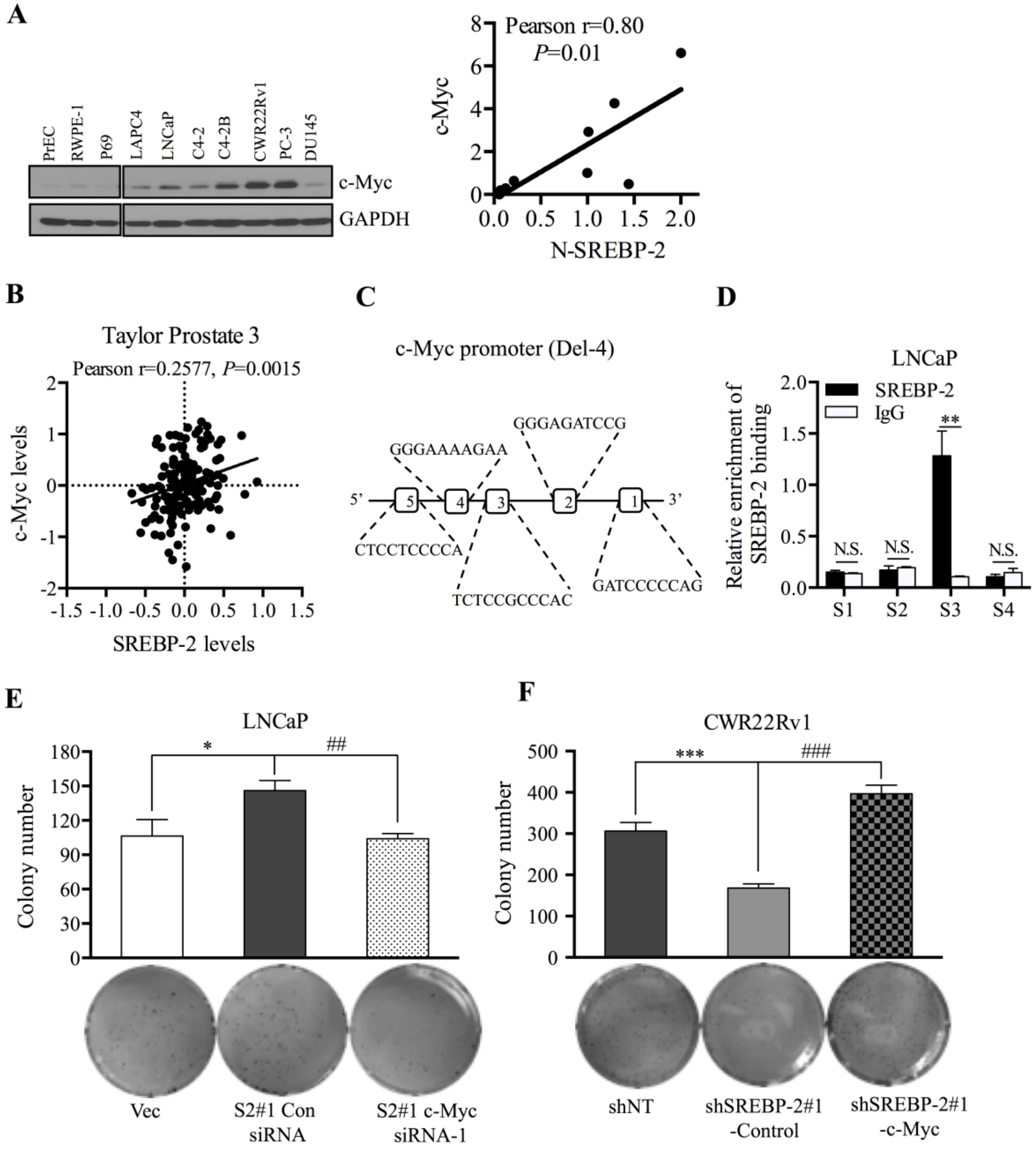

Supplementary Figure S7

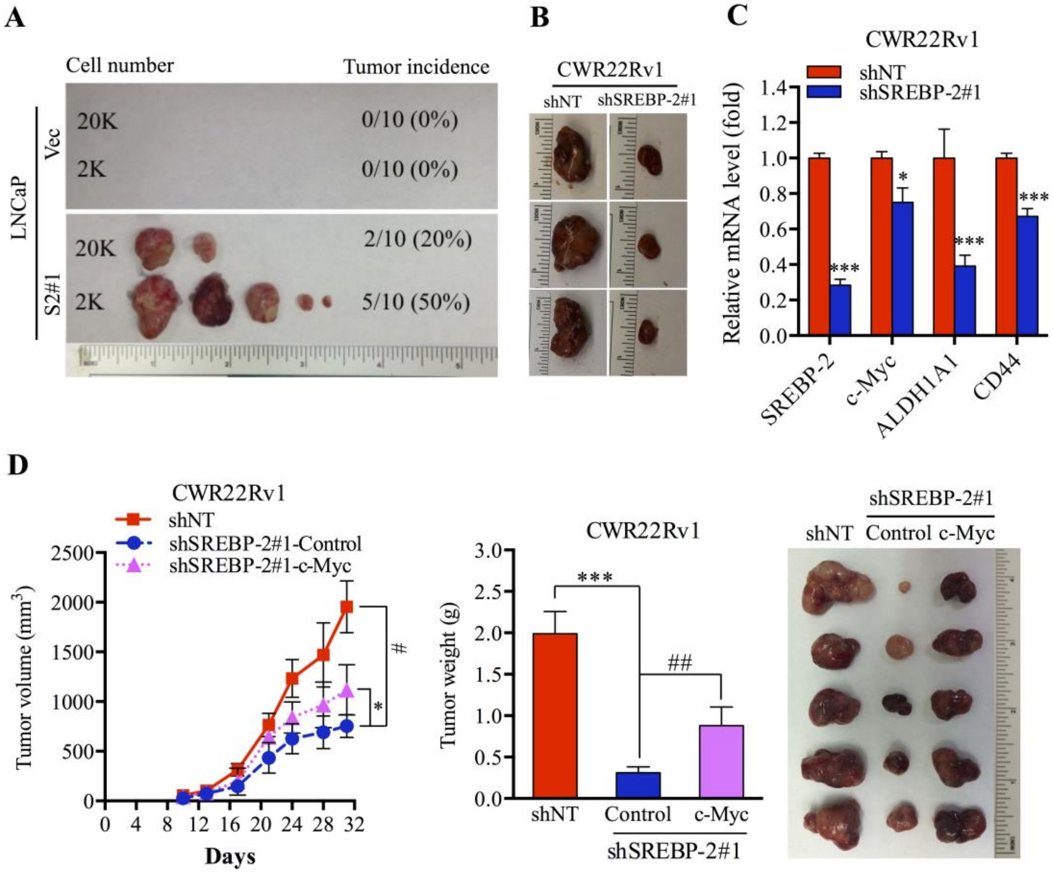

Supplementary Figure S8

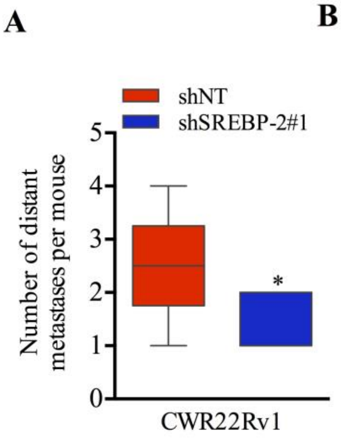

B

Metastasis site	shNT	shSREBP-2#1
Lung	3/8 (37.5%)	0/8 (0%)
Adrenal gland	8/8 (100%)	5/8 (62.5%)
Bone (limbs, jaw, ribs)	5/8 (62.5%)	3/8 (37.5%)

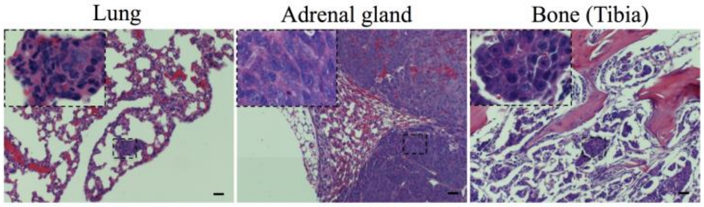

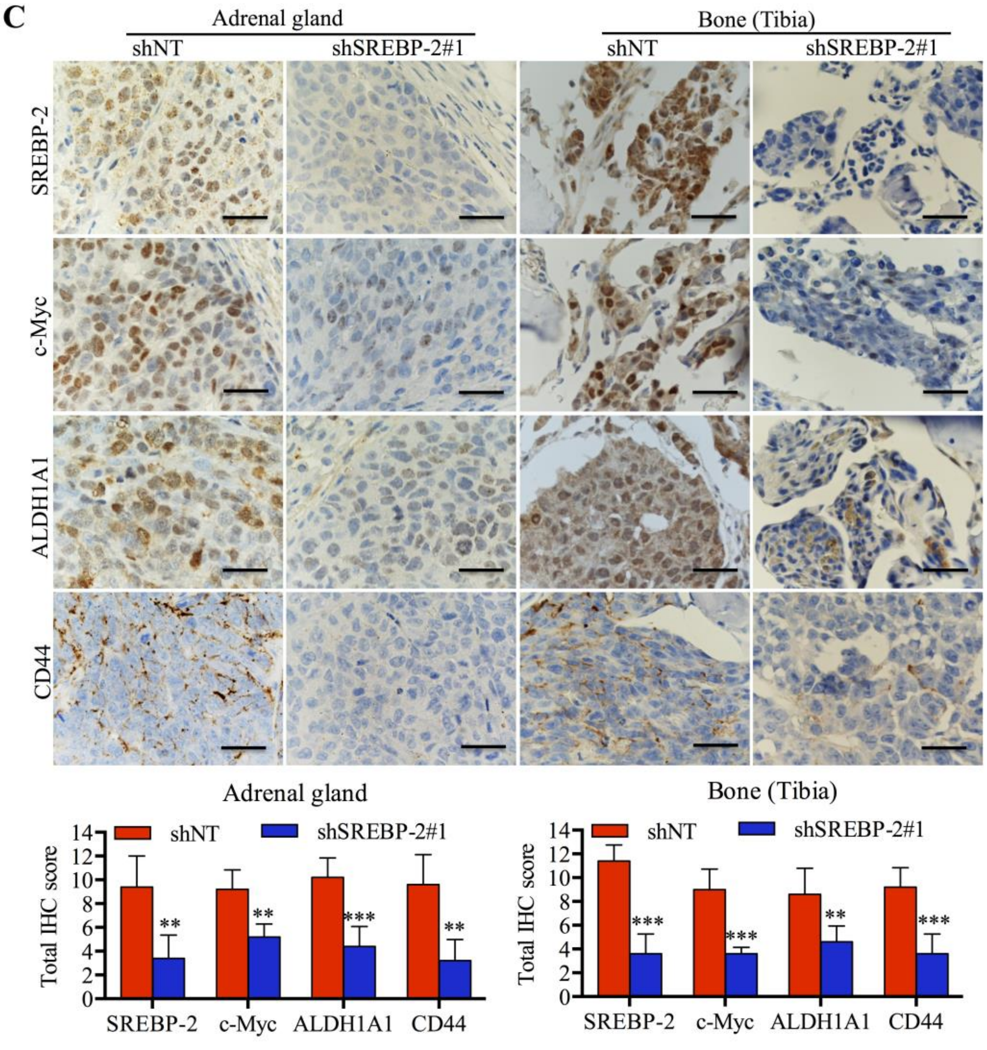

Supplementary Figure S9

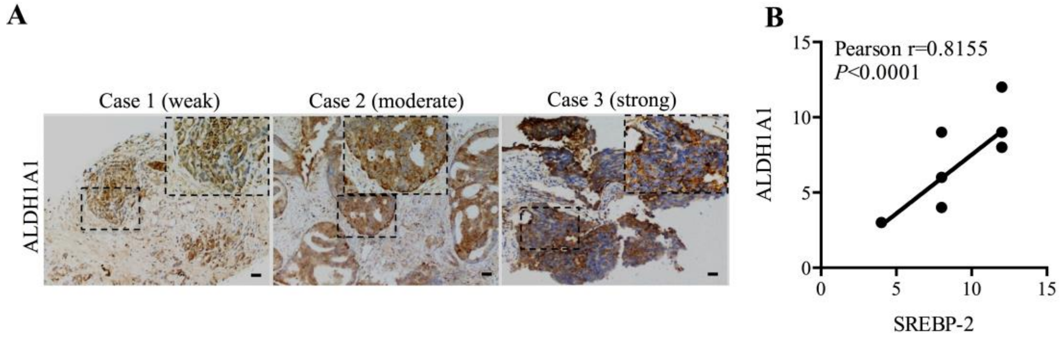

Supplementary Figure S10

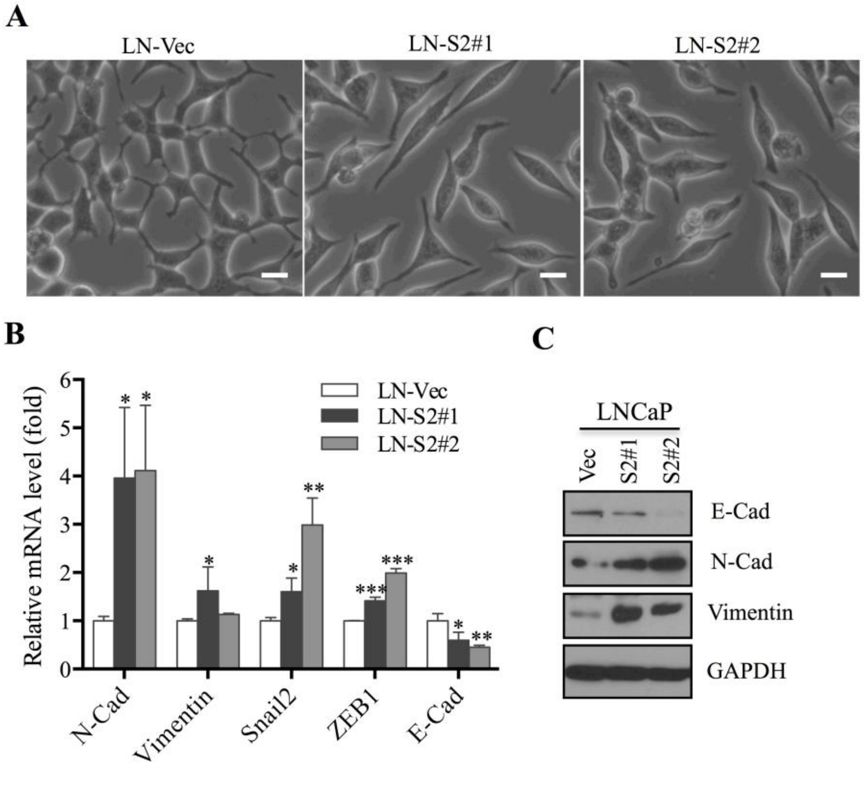

Supplementary Figure S11

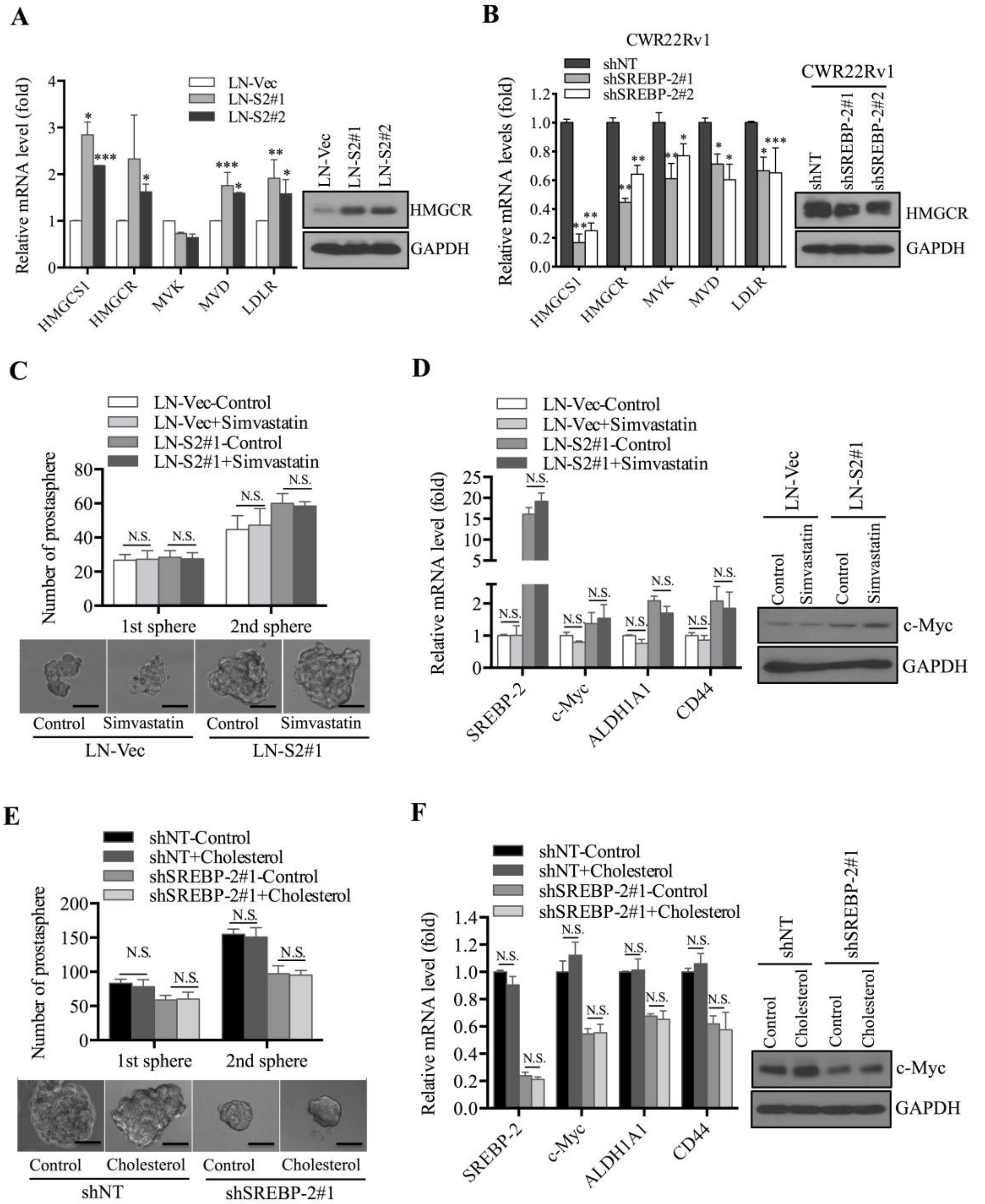

Supplementary Table S1. Clinical and pathological characteristics of human PCa TMA and SREBP-2 expression

No.	Age	Organ	Pathology	TNM	Gleason score	Stage	Clinical Grade	PSA (ng/ml)	SREBP-2 expression
1	59	Bone	Bone carcinoma tissue	M1	10	IV	5	-	+++
2	69	Bone	Small-cell maligent tumor	M1	10	IV	5	-	++
3	61	Bone	Poorly differentation	M1	9	IV	4	-	++
4	65	Bone	Poorly differentation	M1	10	IV	5	-	++
5	44	Prostate	Adenocarcinoma	T3N0M0	6	III	2	-	++
6	63	Prostate	Adenocarcinoma	T3N0M0	8	III	3	13	++
7	53	Prostate	Adenocarcinoma	T4N0M0	7	IV	3	161	+++
8	63	Prostate	Adenocarcinoma	T4N0M0	9	IV	5	91	+++
9	62	Prostate	Adenocarcinoma	T4N0M0	8	IV	4	-	+++
10	75	Prostate	Adenocarcinoma	T3N0M0	8	III	4	98	+
11	70	Prostate	Adenocarcinoma	T3N0M0	8	III	4	0.5	+
12	72	Prostate	Adenocarcinoma	T4N0M0	7	IV	3	16.8	++
13	57	Prostate	Adenocarcinoma	T4N0M0	8	IV	3	25	++
14	63	Prostate	Adenocarcinoma	T4N0M0	9	IV	4	-	+++
15	68	Prostate	Adenocarcinoma	T3N0M0	8	III	4	26.9	++
16	70	Prostate	Adenocarcinoma	T2N0M0	7	II	3	-	++
17	66	Prostate	Adenocarcinoma	T3N0M0	8	III	3	10.8	+
18	72	Prostate	Adenocarcinoma	T3N0M0	9	III	5	-	+++
19	59	Prostate	Adenocarcinoma	T2N0M0	6	II	2	18.3	+
20	70	Prostate	Adenocarcinoma	T3N0M0	7	III	4	14.4	++
21	71	Prostate	Adenocarcinoma	T2N0M0	6	II	2	31.4	++
22	58	Prostate	Adenocarcinoma	T3N0M0	9	III	5	5.8	+++
23	70	Prostate	Adenocarcinoma	T3N0M0	8	III	5	9	+++
24	69	Prostate	Adenocarcinoma	T3N0M0	8	III	4	17.6	++
25	69	Prostate	Adenocarcinoma	T3N0M0	6	III	2	1.1	++
26	67	Prostate	Adenocarcinoma	T3N1M0	9	III	5	13.1	+++
27	65	Prostate	Adenocarcinoma	T3N0M0	9	III	5	17.5	++
28	70	Prostate	Adenocarcinoma	T4N0M0	8	IV	3	7	+++
29	60	Prostate	Adenocarcinoma	T3N0M0	9	III	4	40	+++
30	66	Prostate	Adenocarcinoma	T3N0M0	8	III	5	1.2	+++
31	62	Prostate	Adenocarcinoma	T2N0M0	6	II	2	37.3	+
32	69	Prostate	Adenocarcinoma	T2N0M0	6	II	2	10.6	++
33	73	Prostate	Adenocarcinoma	T2N0M0	6	II	2	48.1	++
34	65	Prostate	Adenocarcinoma	T4N0M0	8	IV	3	34.9	++
35	59	Prostate	Adenocarcinoma	T3N0M0	9	III	4	9.8	+++
36	64	Prostate	Adenocarcinoma	T3N0M0	10	III	5	7.4	+++
37	71	Prostate	Adenocarcinoma	T3N0M0	9	III	5	60.4	+++
38	64	Prostate	Adenocarcinoma	T2N0M0	7	II	3	30	++
39	68	Prostate	Adenocarcinoma	T3N0M0	10	III	5	-	+++

Note: “+” (weak, score 1-4), “++” (moderate, score 5-8) or “+++” (strong, score 9-12).

Supplementary Table S2. Clinical and pathological characteristics of human PCa specimens and the correlation of SREBP-2 with c-Myc or ALDH1A1 expression

No.	Age	Pathology	Metastasis	Gleason score	PSA (ng/ml)	SREBP-2	c-Myc	ALDH1A1
KU1	65	Adenocarcinoma	-	7	10.35	++	+	++
KU2	84	Adenocarcinoma	Bone	7	115.01	+++	+++	++
KU3	77	Adenocarcinoma	Lymph node, bone	8	452.89	++	+++	++
KU4	74	Adenocarcinoma	Lymph node, bone	7	450	++	+++	++
KU5	81	Adenocarcinoma	-	7	8.07	+++	++	+++
KU6	76	Adenocarcinoma	-	7	5.87	+++	++	+++
KU7	61	Adenocarcinoma	-	9	33.77	++	+++	+++
KU8	70	Adenocarcinoma	Lymph node	8	147	+++	+++	+
KU9	77	Adenocarcinoma	Bone	8	206.75	+++	+++	+
KU10	70	Adenocarcinoma	Lymph node, bone	9	90.53	++	+++	++
KU11	81	Adenocarcinoma	Lymph node	9	46.4	+++	+++	++
KU12	70	Adenocarcinoma	Bone	9	28.05	+++	+++	+++
KU13	57	Adenocarcinoma	Bone	9	183.38	++	++	++
KU14	69	Adenocarcinoma	-	9	16.71	+++	+++	+++
KU15	82	Adenocarcinoma	Lymph node, bone	9	1535.4	+++	+++	++
KU16	64	Adenocarcinoma	-	6	-	+	+	+
KU17	71	Adenocarcinoma	-	6	-	+	+	+

Note: “+” (weak, score 1-4), “++” (moderate, score 5-8) or “+++” (strong, score 9-12).

Supplementary Table S3. The correlation of SREBP-2 and c-Myc expression in human PCa tissues

**Number (%)**		**SREBP-2**			χ^{2*}	***P***
		Weak	Moderate	Strong	15.05	0.005
**c-Myc**	Weak	2 (11.8%)	1 (5.9%)	0		
	Moderate	0	1 (5.9%)	2 (11.8%)		
	Strong	0	4 (23.5%)	7 (41.2%)		

Note: weak, score 1-4 (+); moderate, score 5-8 (++); or strong, score 9-12 (+++). * Chi-square test.

Supplementary Table S4. The correlation of SREBP-2 and ALDH1A1 expression in human PCa tissues

**Number (%)**		**SREBP-2**			χ^{2*}	***P***
		Weak	Moderate	Strong	10.98	0.0268
	Weak	2 (11.8%)	0	2 (11.8%)		
**ALDH1A1**	Moderate	0	5 (29.4%)	3 (17.6%)		
	Strong	0	1 (5.9%)	4 (23.5%)		

Note: weak, score 1-4 (+); moderate, score 5-8 (++); or strong, score 9-12 (+++). * Chi-square test.
